# Supplementary material for: A modular toolbox to generate complex polymeric ubiquitin architectures using orthogonal sortase enzymes
Source: Nat Commun. 2021 Nov 11;12:6515. doi: 10.1038/s41467-021-26812-9 (PMC8585875; doi:10.1038/s41467-021-26812-9)
Supplement: Supplementary file 1 — Supplementary Information [file 41467_2021_26812_MOESM1_ESM.pdf]

Supplementary Information

A modular toolbox to generate complex polymeric ubiquitin architectures using orthogonal sortase enzymes

Maximilian Fottner[1,2], Maria Weyh[1], Stefan Gaussmann[3,4], Dominic Schwarz[1], Michael Sattler[3,4] and Kathrin Lang[1,2]*

* to whom correspondence should be addressed: kathrin.lang@org.chem.ethz.ch

[1]Department of Chemistry, Lab for Synthetic Biochemistry, Technical University of Munich, Institute for Advanced Study, TUM-IAS, Lichtenberg Str. 4, 85748 Garching, Germany

[2]Laboratory of Organic Chemistry, Department of Chemistry and Applied Biosciences, ETH Zürich, Vladimir-Prelog-Weg 3, 8093 Zurich, Switzerland

[3]Bavarian NMR Center, Department of Chemistry, Technical University of Munich, Ernst-Otto-Fischer-Str. 2, 85748 Garching, Germany

[4]Institute of Structural Biology, Helmholtz Zentrum München, Ingolstädter Landstrasse 1, 85764 Neuherberg, Germany

Supplementary Figures

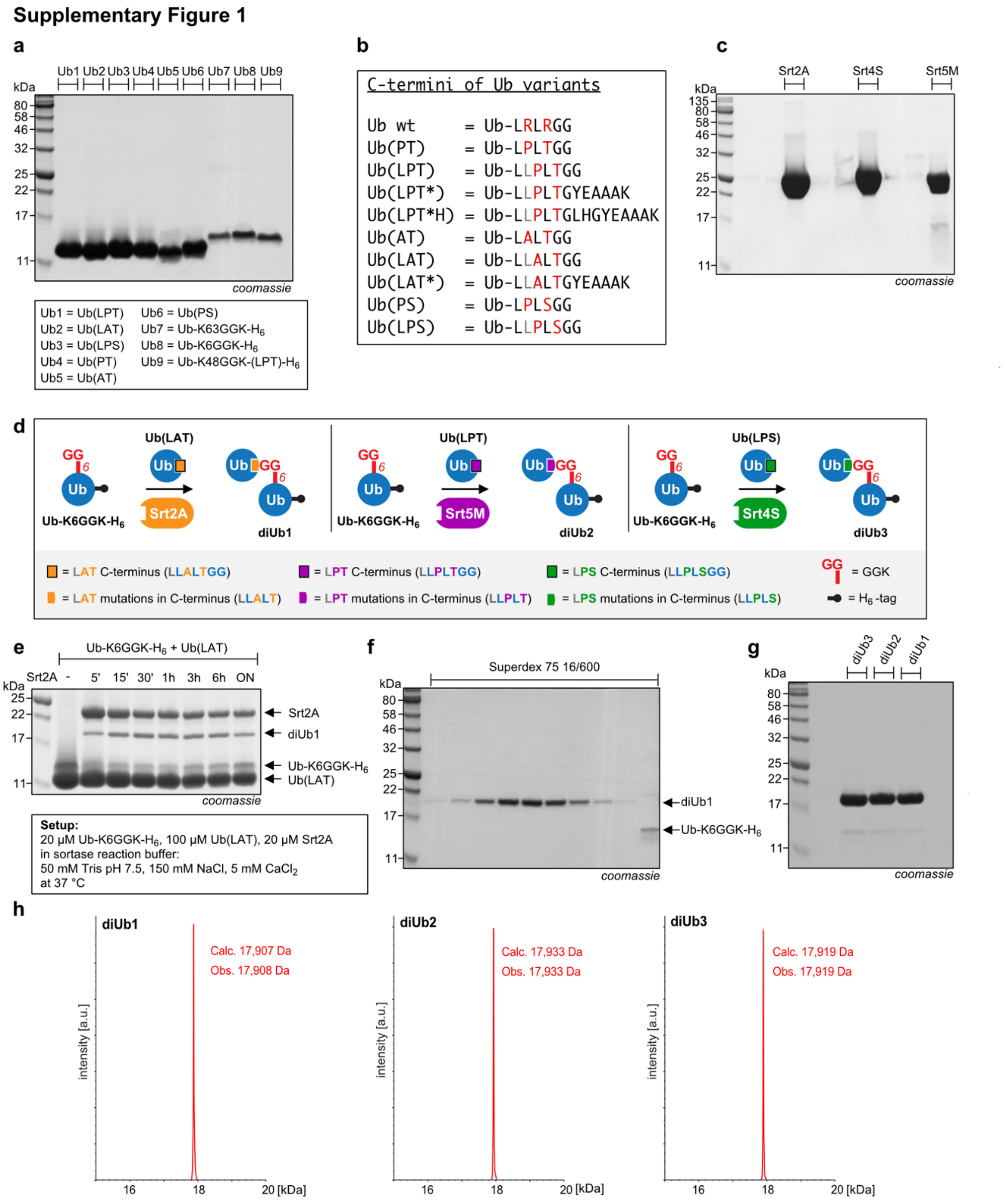

Supplementary Figure 1. Sortase-mediated generation of K6-linked diUbs. a) Overview of purified Ub-variants. **b)** Table displaying different Ub variants used in this study and their respective C-termini. **c)** Overview of purified sortase variants. **d)** Schematic representation for accessing differently linked diUbs, as needed in the diUb hydrolysis assays for identification of orthogonal sortases. **e)** Generation of a K6-linked diUb using Srt2A. Incubation of Ub-K6GGK-H_6 with Ub(LAT) in the presence of Srt2A leads to specific formation of K6-linked diUb (Ub(LAT)-K6-Ub-H_6, diUb1). **f)** Purification of diUbs is carried out by affinity purification (Ni-NTA) and size exclusion chromatography (SEC). Exemplary purification of diUb1 *via* SEC. **g)** Overview of purified K6-linked diUbs with different sortase motifs at the linkage site. **h)** LC-MS characterization of the different K6-linked diUbs.

Yields of sortylation were determined densitometrically using ImageJ from Supplementary Figure 1e: diUb1, 1 h, 61%. Consistent results were obtained over at least three replicate experiments. Source data are provided as a Source Data file.

Supplementary Figure 2

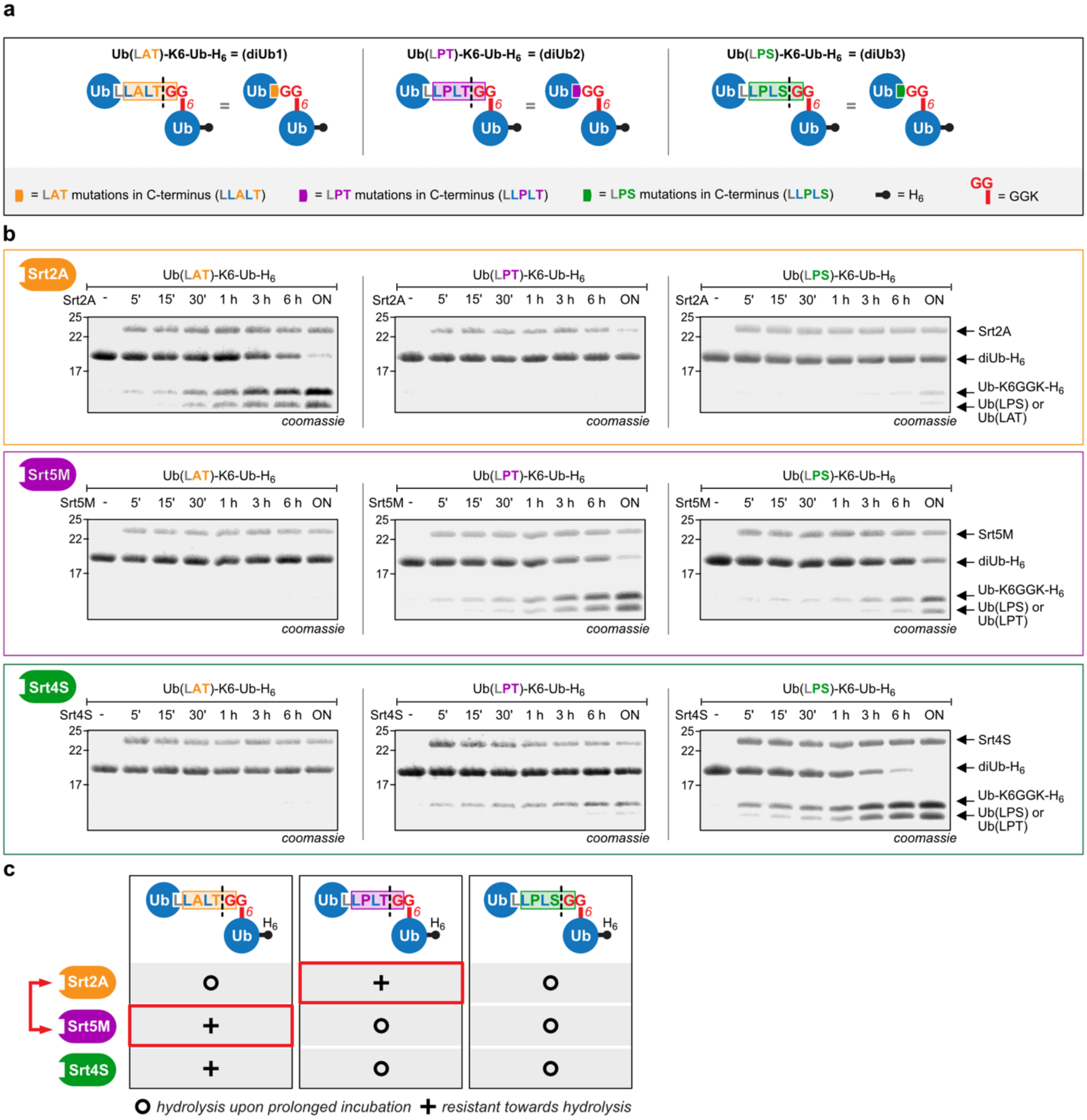

Supplementary Figure 2. Identification of orthogonal sortases *via* hydrolysis of sortase-generated diUbs. a) Overview of sortase-generated diUbs. **b)** Each sortase-generated diUb (diUb1, diUb2 and diUb3) is incubated individually with all three different sortase enzymes. Samples taken at the denoted time points are analysed by SDS-PAGE. All sortase variants exhibit on-target hydrolysis activity on a K6-linked diUb displaying their own recognition motif at the linkage site. Srt4S shows off-target reactivity towards the Srt5M-LPLTG motif, but does not hydrolyse the Srt2A-LALTG linked K6-diUb. Srt5M shows off-target reactivity towards the Srt4S-LPLSG motif, but does not hydrolyse the Srt2A-LALTG motif. Srt2A shows weak off-target reactivity for the Srt4S-LPLSG motif and is not able to hydrolyse the Srt5M-LPLTG motif. As Srt2A and Srt5M do not hydrolyse/recognize their respective recognition motifs, Srt2A/Srt5M represent a pair of bidirectional orthogonal sortases. **c)** Schematic representation of hydrolysis assay. Consistent results were obtained over at least three replicate experiments. Source data are provided as a Source Data file.

Supplementary Figure 3

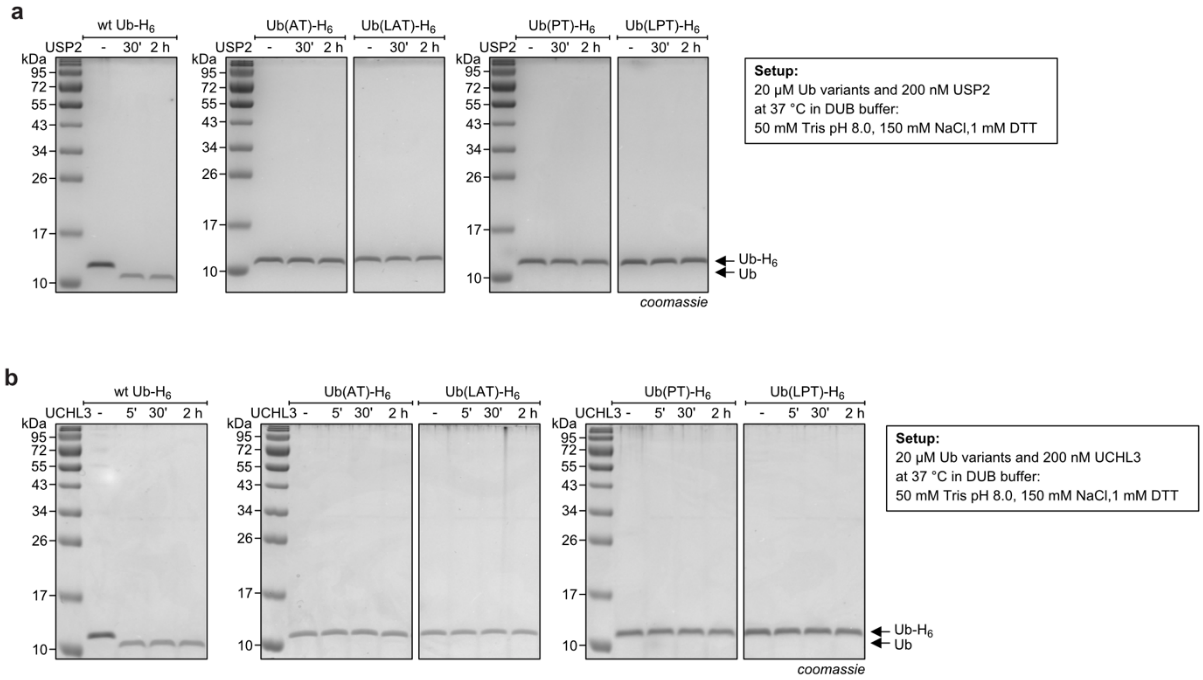

Supplementary Figure 3. DUB assays with Ub variants bearing different C-terminal mutations. a) SDS-PAGE analysis of DUB hydrolysis assays with the catalytic domain of USP2. In presence of USP2, wt Ub with a C-terminal H_6-tag shows quantitative H_6-tag cleavage within 30 min. With Ub variants bearing the different sortase motifs (AT, LAT, PT, LPT) at their C-termini, H_6-tag cleavage was not observed. **b)** SDS-PAGE analysis of DUB assays with UCHL3. In presence of UCHL3, wt Ub with a C-terminal H_6-tag shows quantitative H_6-tag cleavage within 5 min. With Ub variants bearing the different sortase motifs (AT, LAT, PT, LPT) at their C-termini, H_6-tag cleavage was not observed. Consistent results were obtained over at least three replicate experiments. Source data are provided as a Source Data file.

Supplementary Figure 4

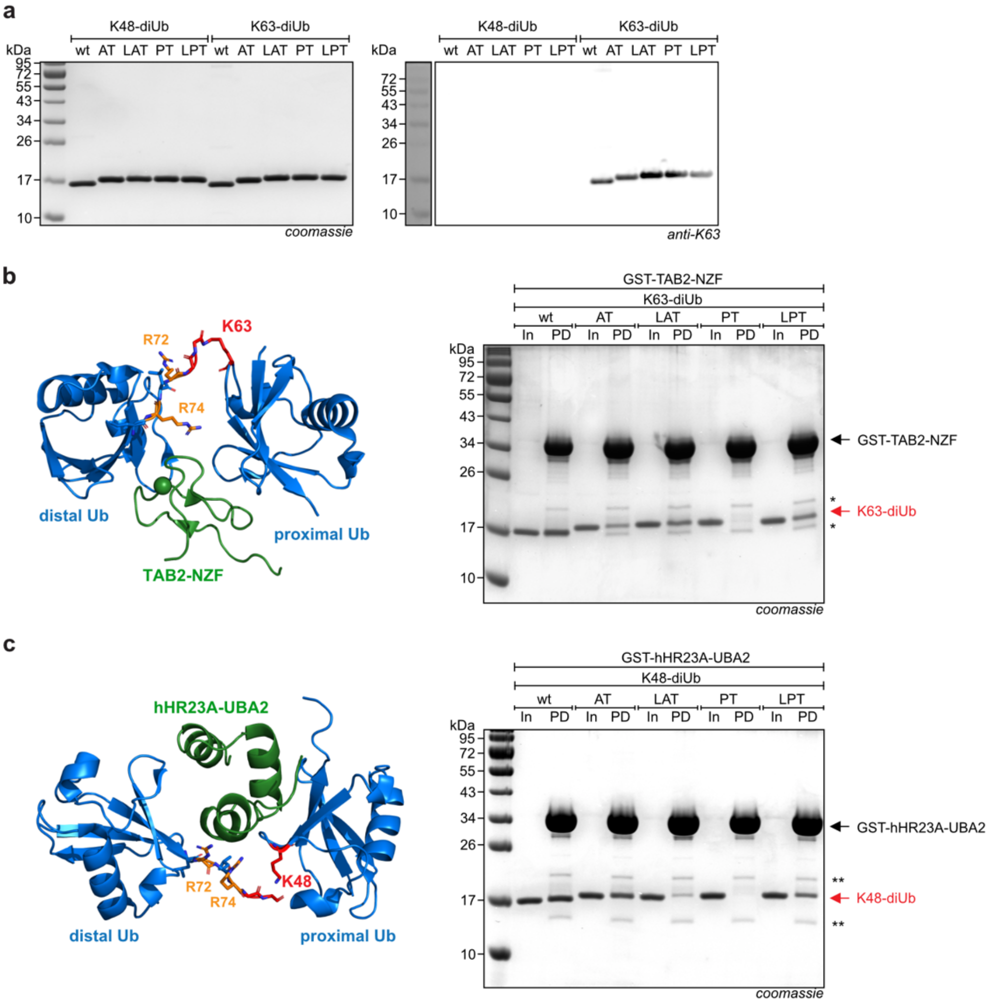

Supplementary Figure 4. Interaction of sortase-generated diUbs with Ub-binding domains (UBDs) a) Probing different K48- and K63-linked diUbs displaying different sortase motifs with a K63-specific antibody. Left: Coomassie-stained SDS PAGE gel, right: Western blot with an anti-K63-linkage specific antibody. **b)** Interaction of differently linked K63-diUbs with TAB2-NZF. Left: Structure of K63-diUb bound to TAB2-NZF (PDB: 2WWZ[1]). Right: Full SDS-PAGE of pull-down (PD) experiments with differently linked K63-diUbs and GST-fused TAB2-NZF. **c)** Interaction of differently linked K48-diUbs with hHR23A UBA2. Left: NMR structure of the hHR23A UBA2 domain bound to K48-linked diUb (PDB: 1ZO6[2]). Right: Full SDS-PAGE of pull-down experiments with differently linked K48-diUbs and GST-fused hHR23A-UBA2. In: input sample i.e. differently linked K48- or K63-diUbs, PD: pull-down. '*' marks impurities present in GST-TAB2-NZF. '**' marks impurities present in GST-hHR23A-UBA2. Consistent results were obtained over at least three replicate experiments. Source data are provided as a Source Data file.

Supplementary Figure 5

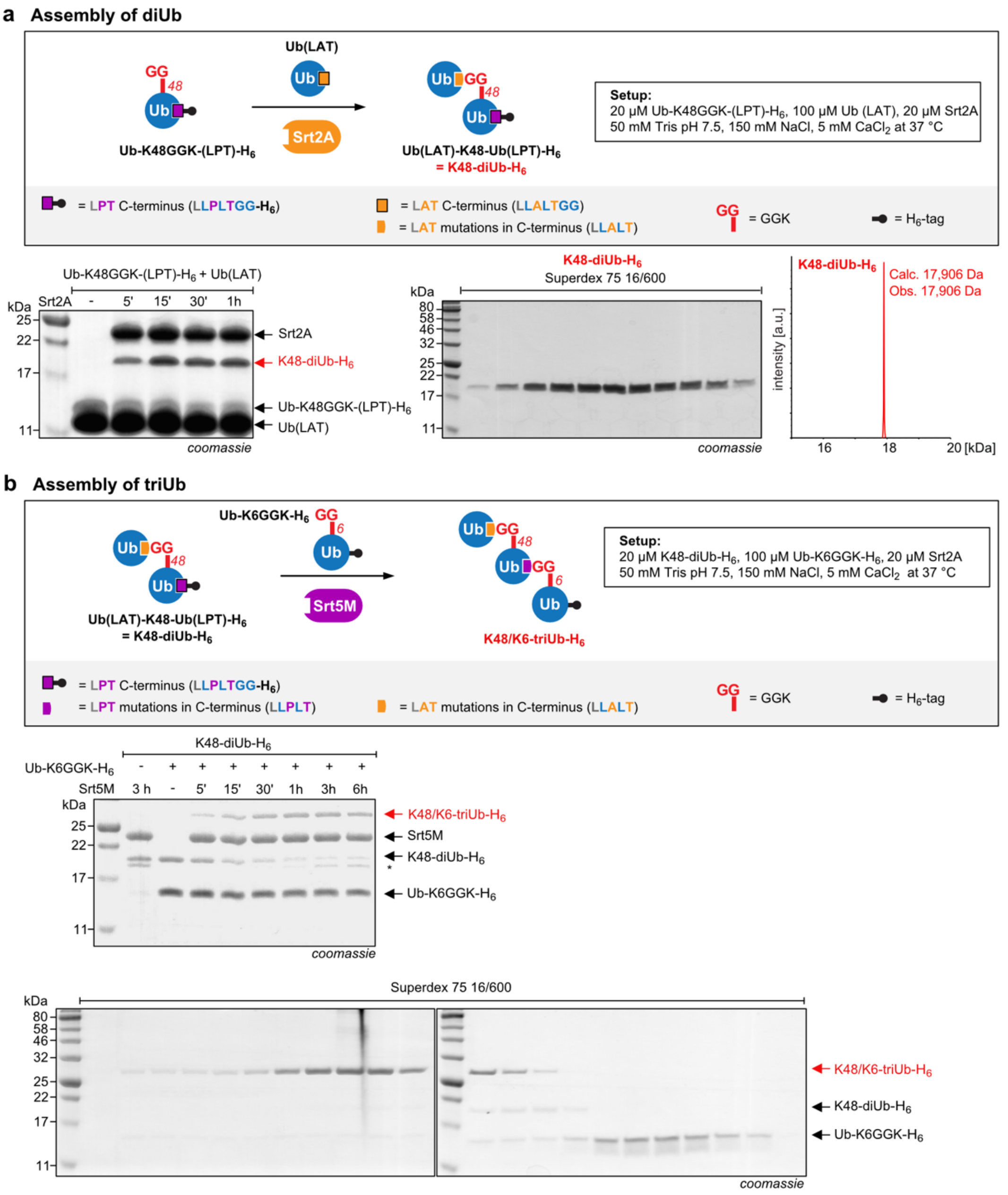

Supplementary Figure 5. Generation of a heterotypically linked triUb using the orthogonal sortase pair Srt2A/Srt5M. a) Schematic representation of the Srt2A-mediated assembly of K48-diUb as needed for subsequent triUb formation. SDS-PAGE analysis displaying that incubation of Ub-K48GGK-(LPT)-H_6 with Ub(LAT) in the presence of Srt2A leads to formation of Ub(LAT)-K48-Ub-(LPT)-H_6, denoted as K48-diUb-H_6. K48-diUb-H_6 was purified *via* Ni-NTA (Ni-Nitrilotriacetic acid) chromatography and SEC (Superdex 75 16/600). LC-MS analysis confirmed the identity of the Srt2A-generated K48-diUb-H_6. **b)** Schematic representation of the Srt5M-mediated assembly of the heterotypically linked K48/K6 triUb. SDS-PAGE analysis shows Srt5M-catalyzed transpeptidation between the K48-diUb-H_6 and Ub-K6GGK-H_6 resulting in the formation of heterotypically K48/K6 linked triUb (K48/K6-triUb-H_6). '*' denotes Srt5M-mediated hydrolysis of the Srt5M recognition motif and thereby cleavage of H_6-tag from K48-diUb-H_6. K48/K6-triUb was purified by Ni-NTA chromatography and SEC (Superdex 75 16/600).

Yields of sortylation were determined densitometrically using ImageJ from: Supplementary Figure 5a: K48-diUb(LAT)-H_6, 30', 63%; Supplementary Figure 5b: K48/K3-triUb-H_6, 3 h, 78%. Consistent results were obtained over at least three replicate experiments. Source data are provided as a Source Data file.

Supplementary Figure 6

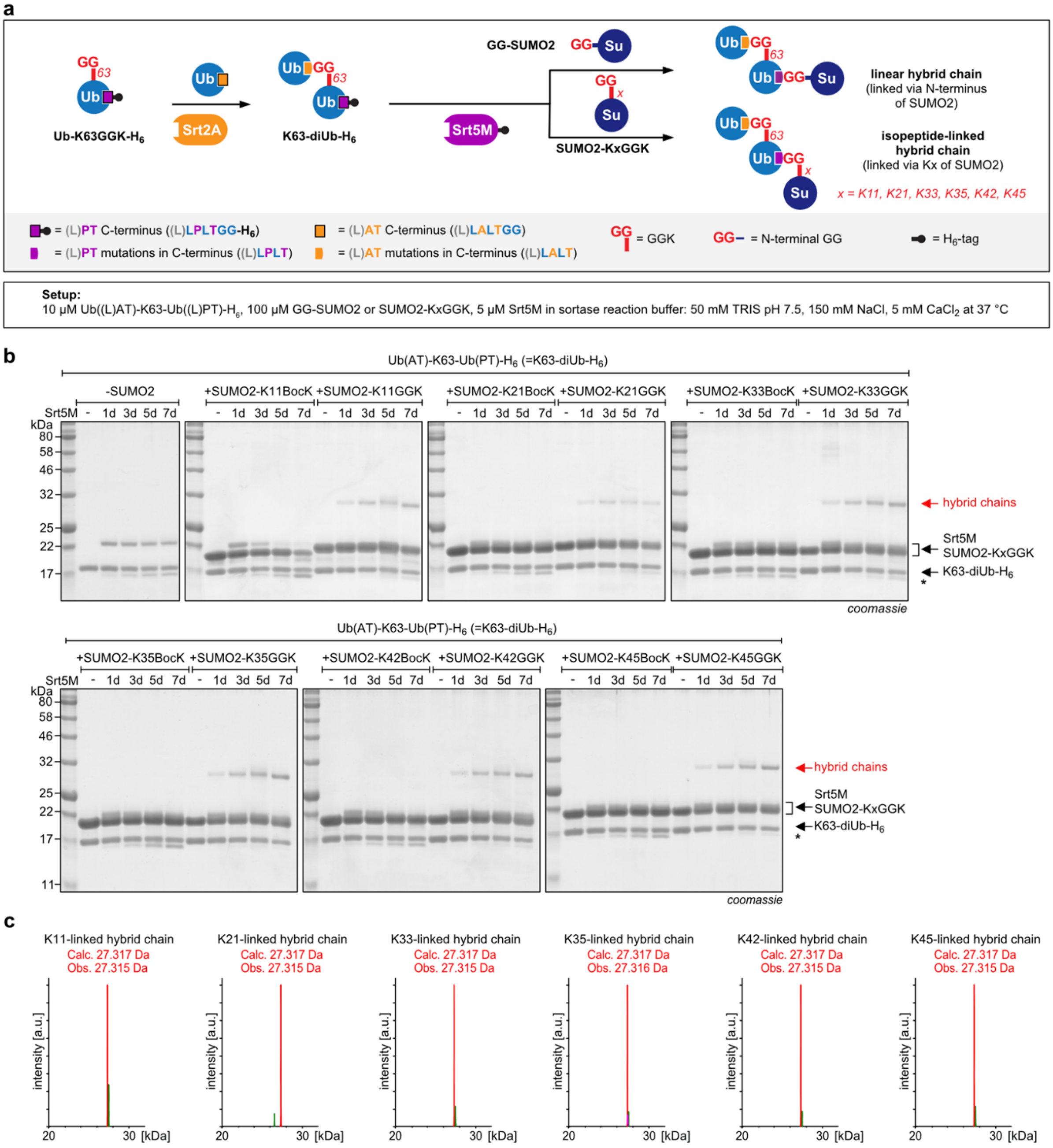

Supplementary Figure 6. **Generation of differently linked K63-diUb-SUMO2 hybrid chains using the orthogonal Srt2A/Srt5M pair. a)** Schematic representation of the two-step assembly of differently linked K63-diUb-SUMO2 hybrid chains using Srt2A and Srt5M (either with spacer leucine or without). Srt2A-generated K63-linked diUb is incubated with SUMO2 variants bearing a GGK moiety at K11, K21, K33, K35, K42 or K45 for generating isopeptide-linked hybrid chains or with a SUMO2 variant bearing an N-terminal GG-sequence for building a linearly-linked hybrid chain. **b)** SDS-PAGE analysis of formation of differently isopeptide-linked hybrid chains displaying AT and PT linkages. Srt2A-generated K63-diUb-H_6 (Ub(AT)-K63-Ub(PT-H_6) is incubated with SUMO2-KxGGK or SUMO2-KxBocK variants in the presence of Srt5M. Hybrid chain formation is dependent on the presence of the acceptor nucleophile GGK in SUMO2-KxGGK. In absence of SUMO2-KxGGK, hydrolysis of the Srt5M recognition motif and thereby cleavage of H_6-tag of K63-diUb-H_6 is observed (denoted by '*'). **c)** LC-MS analysis confirmed the integrity of the differently linked hybrid chains.

Yields of sortylation were determined densitometrically using ImageJ from Supplementary Figure 6b: K11, 7 d, 35 %; K21, 7 d, 16 %; K33, 7 d, 41 %; K35, 7 d, 49 %; K42, 7 d; 44 %, K45, 7 d, 42 %.

Consistent results were obtained over at least three replicate experiments.

Supplementary Figure 7

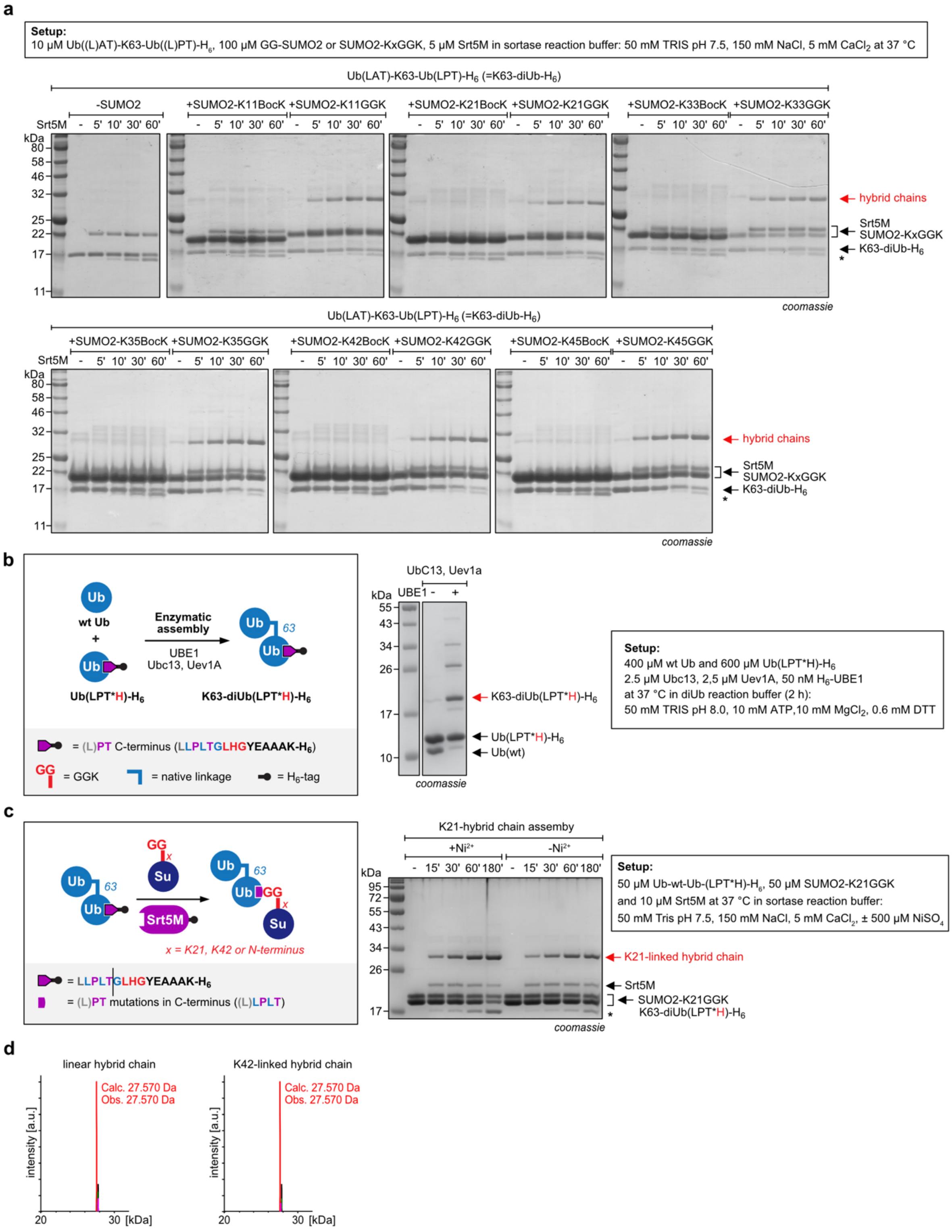

Supplementary Figure 7. Generation of differently linked K63-diUb-SUMO2 hybrid chains *via* the orthogonal Srt2A/Srt5M pair and enzymatic assembly. a) SDS-PAGE analysis of formation of differently isopeptide-linked hybrid chains. Srt2A-generated (Ub(LAT)-K63-Ub(LPT)-H_6) is incubated with SUMO2-KxGGK or SUMO2-KxBocK variants in the presence of Srt5M. Hybrid chain formation is dependent on the presence of the acceptor nucleophile GGK in SUMO2-KxGGK. In absence of SUMO2-KxGGK, hydrolysis of the Srt5M recognition motif and thereby cleavage of H_6-tag of K63-diUb-H_6 is observed (denoted by '*'). **b)** Enzymatic assembly of natively linked K63-diUb. Ub wt is incubated with a Ub-variant bearing the Srt5M recognition motif (LLPLTG) lacking the C-terminal G76, followed by a short linker (LHGYEAAAK; dubbed

Ub(LPT*H)-H_6), in presence of the E1-enzyme UBE1 and the E2-enzymes Ubc13 and Uev1A. Ub(LPT*H)-H_6 lacks G76 thereby guaranteeing orthogonality towards E1/E2-enzymes. Additionally, Ub(LPT*H)-H_6 bears a GLHG motif that facilitates subsequent selective nucleophile quenching *via* Ni^{2+} complexation to restrict the reversibility of the ligation reaction[3]. **c)** Assembly of diUb-SUMO2 hybrid chains containing the wt linker between the Ub moieties. Equimolar ratios of K63-diUb and SUMO2-K21GGK are incubated with Srt5M either in presence or absence of Ni^{2+}. Selective nucleophile quenching increases hybrid chain yield by 1.5-fold when using equimolar concentration of diUb and SUMO2-GGK. (yields were determined densitometrically using ImageJ software (Wayne Rasband, National Institutes of Health, USA, http://imagej.nih.gov/ij). **d)** LC-MS analysis confirmed the identity of the linear and K42-linked hybrid chains.
Yields of sortylation were determined densitometrically using ImageJ from:
Supplementary Figure 7a: K11, 1 h, 55 %; K21, 1 h, 47 %; K33, 1 h, 56 %; K35, 1 h, 63 %; K42, 1 h; 65 %, K45, 1 h, 62 %.
Supplementary Figure 7c: +Ni^{2+}, 3 h, 39 %; -Ni^{2+}, 3 h, 28 %.
Consistent results were obtained over at least three replicate experiments.
Source data are provided as a Source Data file.

Supplementary Figure 8

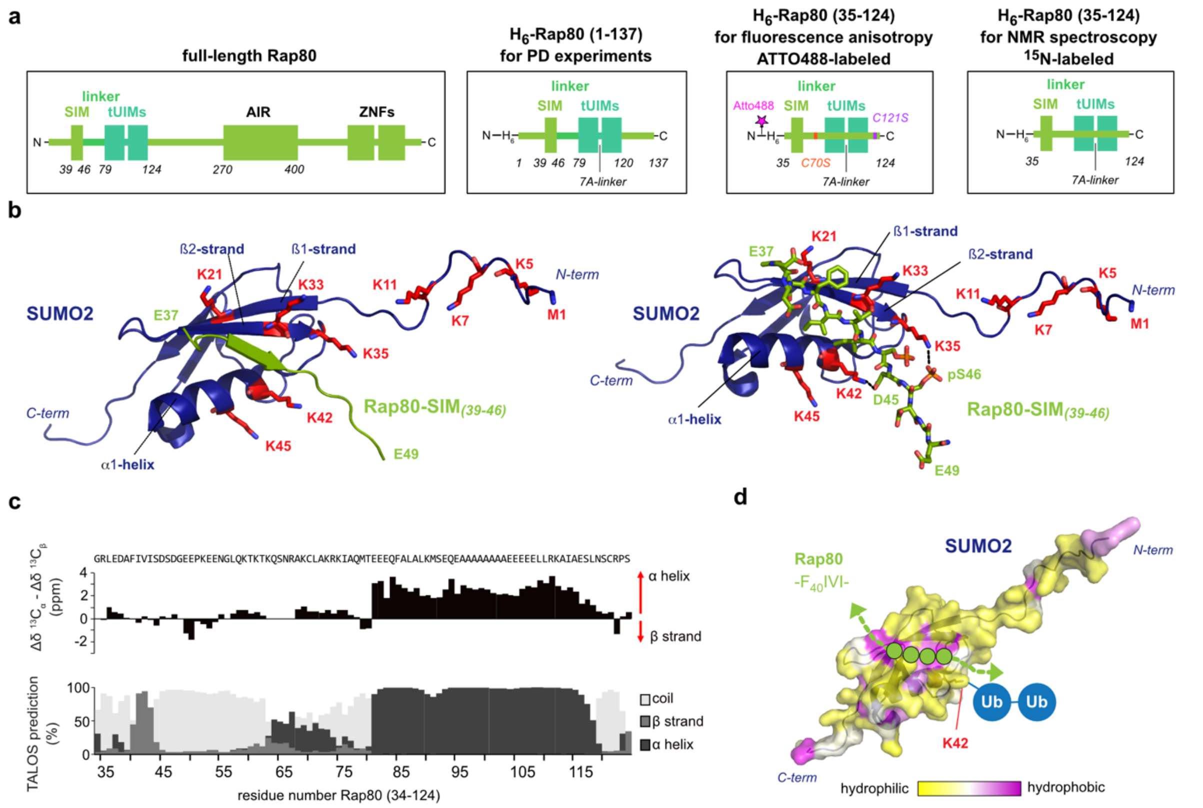

Supplementary Figure 8. Rap80 engagement with K63-diUb-SUMO2 hybrid chains. a) Overview of different Rap80 constructs used in this study. Rap80 is a 719-residue multidomain protein consisting of an N-terminal SUMO interacting motif (SIM ~residues 39-46) and tandem Ub-interacting motifs (tUIMs, ~residues 79-120) as well as a C-terminal part harbouring an Abraxas interacting region (AIR) and two putative zing fingers (ZNF). An N-terminally tagged Rap80 construct (residues 1-137) was used for pull-down (PD) experiments. It contains a 7A-linker between the two tUIMs (residues 97-103)[4]. For anisotropy experiments, the construct was shortened (residues 35-124), C70/C121 were mutated to serine and a cysteine was introduced N-terminally to the H_6-tag for labelling with Atto488 maleimide. For NMR studies the Rap80$_{(35-124)}$ construct was used. **b)** Structural insights into the interaction between the N-terminal SIM of Rap80 and SUMO2. The flexible N-terminal region of SUMO2 harbouring lysine residues K5, K7 and K11 is not involved in binding of the SIM of Rap80. Interaction mainly occurs in a defined hydrophobic binding groove located between the α_1 helix and the β_2 strand of the SUMO2 core. Lysine residues K33 (electrostatic interactions), K35 (interaction with Ser/pSer residues 44 and 46 of Rap80) and K42 (interaction with D45 of Rap80) were shown to be essential for the interaction with the SIM of Rap80. In contrast, lysine residues K21 (located in the β_1 strand) and K45 of the SUMO2 core do not seem to be directly involved in the binding of the SIM of Rap80. The scheme is based on PDB: 2N9E2[5]. **c)** ^{13}C secondary chemical shifts ($\Delta\delta^{13}C_\alpha$ - $\Delta\delta^{13}C_\beta$) (top panel) and TALOS+[6] secondary structure prediction from shifts (bottom panel) indicate a helical propensity for residues 62-79 preceding the performed α-helical conformation of the tandem IUM including the 7A linker. **d)** Schematic representation of the parallel and anti-parallel binding mode of the SIM core motif F$_{40}$IVI, represented as arrows, into the hydrophobic pocket (magenta) of SUMO2. K63-diUb linkage at K42 abolishes K42-D54 and K35-S46 interactions and sterically reduces the accessibility of the hydrophobic groove which hinders binding of Rap80 SIM in the preferred parallel conformation and leads to a small occupancy of the non-favoured antiparallel binding. This scheme is based on PDB: 2N9E[5]. Surface was coloured using hydrophobicity scale from Kyte and Doolittle[7].

Supplementary Figure 9

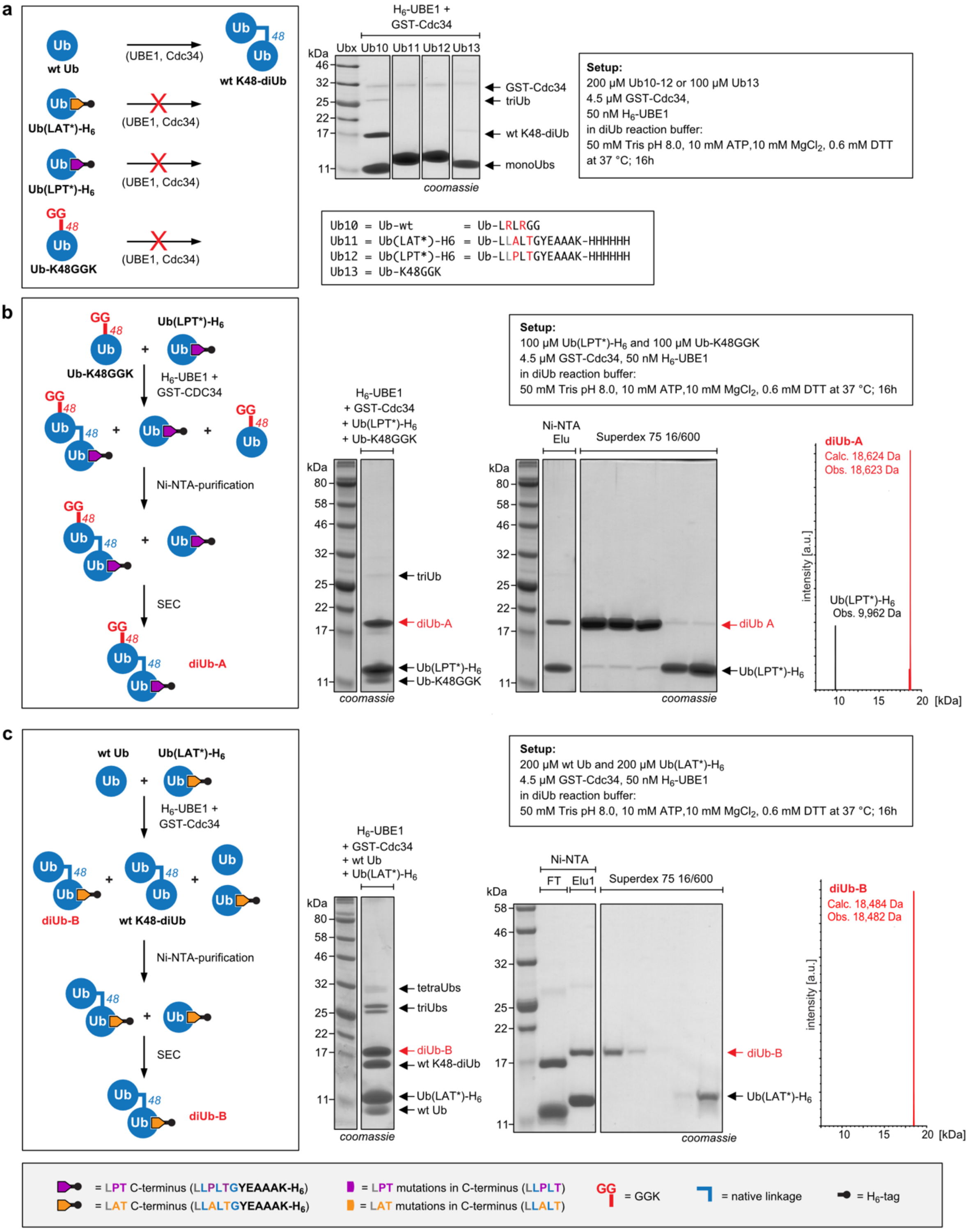

Supplementary Figure 9. Enzymatic assembly of K48-linked diUbs for subsequent K48-tetraUb formation. a) Overview of Ub buildings blocks used for the generation of diUb-A and diUb-B. Schematic representation (left) and SDS-PAGE analysis (right) of enzymatic K48-diUb formation using the E1 Ub-activating enzyme (UBE1) and the K48-linkage specific E2 Ub-conjugating enzyme (CDC34) and distinctly modified Ub monomers. Wt Ub shows di/tri Ub formation when incubated with UBE1 and CDC34, as both the C-terminus and K48 are accessible for Ub-conjugation. In contrast, Ub(LAT*)-H_6 and Ub(LPT*)-H_6 lack G76 at the C-terminus and display an additional YEAAAK sequence, rendering them ineffective for E1/E2-mediated enzymatic assembly to form K48-linked polyUbs. Ub-K48GGK bears GGK at K48 making it conjugation incompetent for enzymatic assembly of polyUbs using UBE1 and the K48-linkage specific CDC34. **b)** Enzymatic assembly and purification of diUb-A. SDS-PAGE analysis of overnight incubation of Ub(LPT*)-H_6 and Ub-K48GGK in presence of UBE1 and CDC34

leads to formation of K48-linked diUb (with a minute amount of K48-triUb). Purification of diUb-A is performed using Ni-NTA chromatography followed by SEC (Superdex 75 16/600). LC-MS analysis confirms identity of diUb-A, but also shows that diUb-A contains residual Ub(LPT*)-H_6 as impurity, which however cannot participate in the subsequent Srt2A-catalzyed transpeptidation reaction. **c)** Enzymatic assembly and purification of diUb-B. SDS-PAGE analysis of overnight incubation of wt Ub and Ub(LAT*)-H_6 in the presence of UBE1 and CDC34 leads to formation of K48-linked di- and triUb species. Purification of diUb-B is performed by Ni-NTA chromatography followed by SEC (Superdex 75 16/600). LC-MS analysis confirms identity of diUb-B. Ni-NTA: Ni-Nitrilotriacetic acid, FT: flow-through, Elu: eluant. Consistent results were obtained over at least three replicate experiments. Source data are provided as a Source Data file.

Supplementary Figure 10

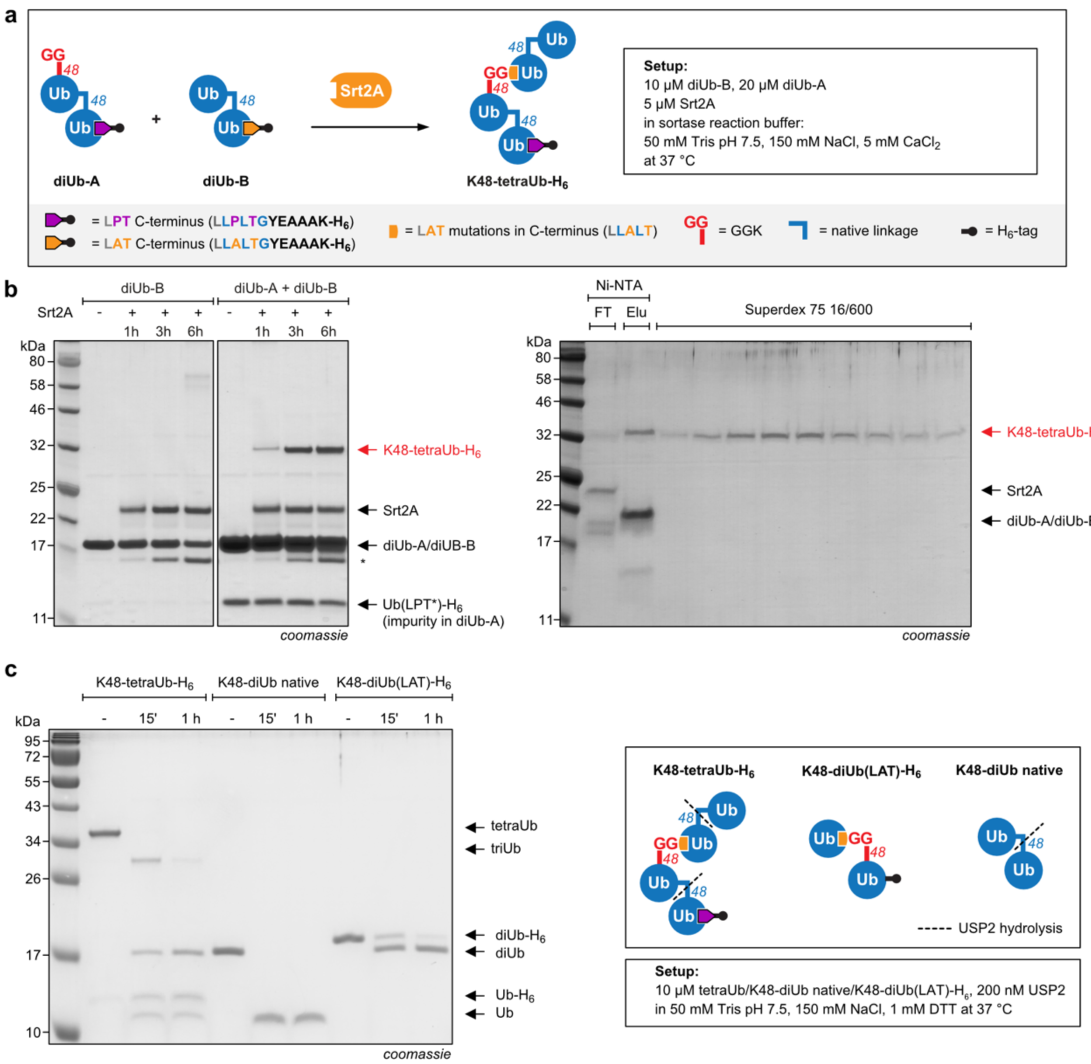

Supplementary Figure 10. Sortase-mediated generation of a K48-linked tetraUb and hydrolysis assay with USP2. a) Schematic representation of Srt2A-mediated tetraUb formation using enzymatically accessed diUb-A and diUb-B. **b)** Left: Incubation of diUb-A and diUb-B in presence of Srt2A leads to the formation of K48-linked tetraUb. TetraUb formation cannot be observed in the absence of diUb-B, confirming the orthogonality of Srt2A towards the Srt5M-recognition motif LPLTG. '*' denotes the hydrolysis product of the Srt2A- motif at the C-terminus of diUb-B in the absence of a GGK-acceptor nucleophile and thereby cleavage of the H_6-tag. Right: Purification of K48-linked tetraUb is performed *via* Ni-NTA chromatography followed by SEC (Superdex 75 16/600). Ni-NTA: Ni-Nitrilotriacetic acid, FT: flow-through, Elu: Eluant. **c)** USP2 hydrolysis assays of K48-tetraUb-H_6, wt K48-diUb and K48-diUb(LAT)-H_6. Left: Treatment of sortase-generated K48-tetraUb-H_6 with USP2 yields K48-diUb(LAT), Ub(LPT)-H_6 and Ub(wt) as cleavage products. As expected, the 'LAT/LPT' linkages are recalcitrant towards cleavage by USP2. Control assays, in which K48-diUb native is treated with USP2 result in the formation of Ub(wt). Treatment of K48-diUb(LAT)-H_6 with USP2 leads to cleavage of the H_6-tag of the proximal Ub, while the 'LAT' diUb linkage is resistant towards DUB hydrolysis.
Yields of sortylation were determined densitometrically using ImageJ from: Supplementary Figure 10b: K48-tetraUb, 6 h, 37 %.
Consistent results were obtained over at least three replicate experiments.
Source data are provided as a Source Data file.

Supplementary Figure 11

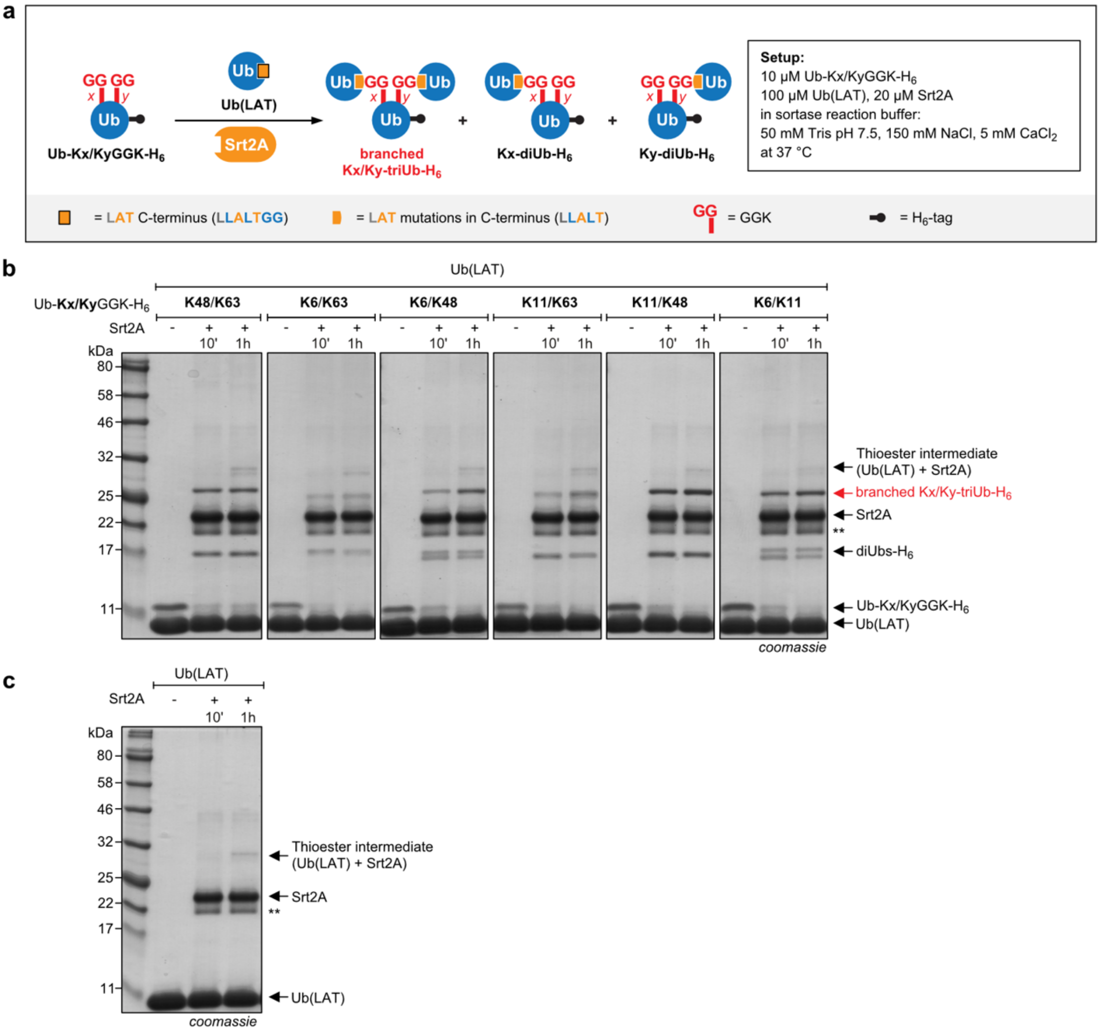

Supplementary Figure 11. Generation of differently branched triUbs. a) Schematic representation of sortase-mediated assembly of branched triUbs *via* incubation of Ub-Kx/KyGGK-H_6 with Ub(LAT) in the presence of Srt2A. **b)** SDS-PAGE analysis of incubation of different Ub-Kx/KyGGK-H_6 variants with Ub(LAT) and Srt2A shows formation of desired branched triUbs as well as of both diUb intermediates. '**' denotes an impurity present in Srt2A. **c)** Incubation of Ub(LAT) with Srt2A does not lead to di/triUb formation in the absence of a GGK-modified Ub. A slight band for Srt2A-Ub(LAT) thioester intermediate formation can be observed. '**' denotes an impurity present in Srt2A.

Yields of sortylation were determined densitometrically using ImageJ from:

Supplementary Figure 11b: K48/K63, 1 h, 43 %; K6/K63, 1 h, 53 %; K6/K48, 1 h, 47 %; K11/K63, 1 h, 54 %; K11/K48, 1 h, 51 %; K6/K11, 1 h, 51 %.

Consistent results were obtained over at least three replicate experiments.

Source data are provided as a Source Data file.

Supplementary Figure 12

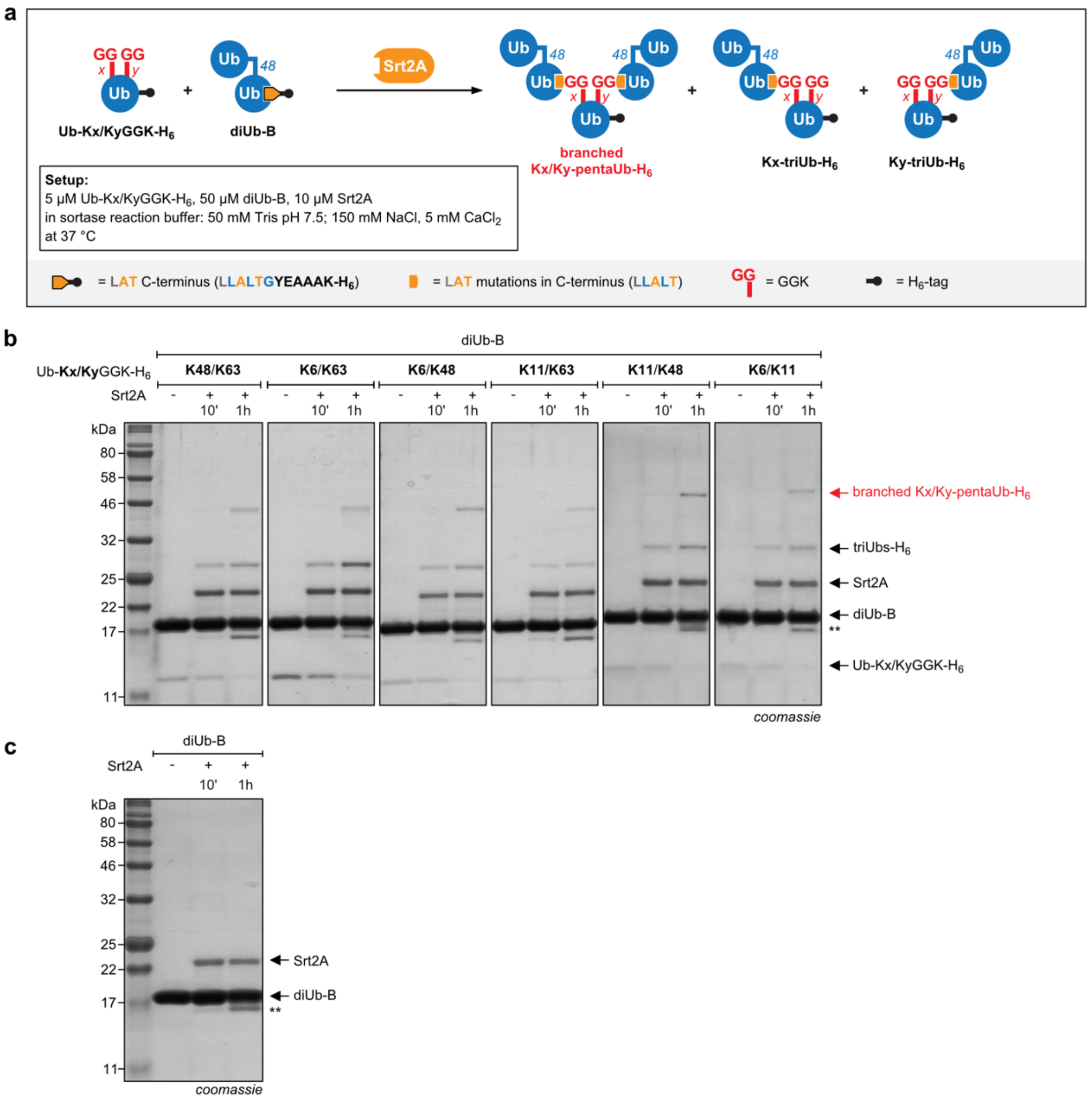

Supplementary Figure 12. Generation of differently branched pentaUbs. a) Schematic representation of sortase-mediated assembly of branched pentaUbs. **b)** Incubation of various Ub-Kx/KyGGK-H_6 with diUb-B in the presence of Srt2A leads to formation of the desired branched pentaUb and both triUb intermediates, as shown by SDS-PAGE analysis. '**' denotes impurity present in Srt2A preparation. **c)** Incubation of diUb-B with Srt2A in the absence of GGK-modified Ub does not lead to tri/pentaUb formation. '**' denotes impurity present in Srt2A.
Yields of sortylation were determined densitometrically using ImageJ from:
Supplementary Figure 12b: K48/K63, 1 h, 35 %; K6/K63, 1 h, 19 %; K6/K48, 1 h, 39 %; K11/K63, 1 h, 38 %; K11/K48, 1 h, 41 %; K6/K11, 1 h, 27 %.
Consistent results were obtained over at least three replicate experiments.
Source data are provided as a Source Data file.

Supplementary Figure 13

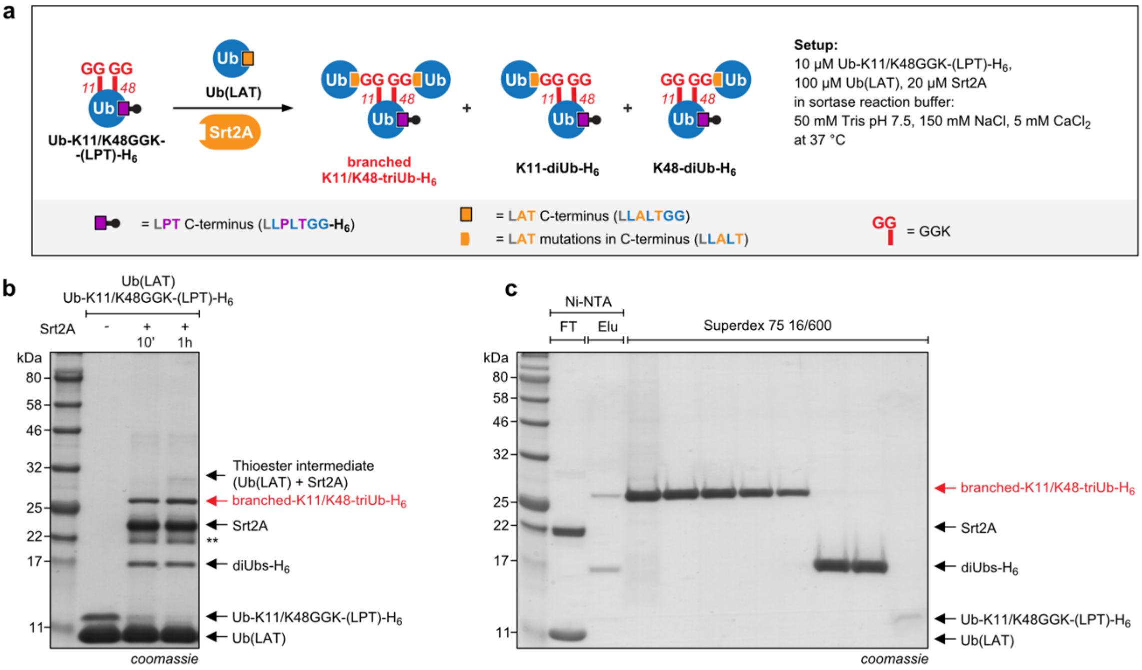

Supplementary Figure 13. Generation and purification of the K11/K48-branched triUb. a) Schematic representation of sortase-mediated assembly of the K11/K48 branched triUb used for POI charging. To site-specifically attach the triUb onto a POI, the starting Ub-Kx/KyGGK-H_6 building block was equipped with a C-terminal Srt5M recognition motif. **b)** Incubation of Ub-K11/K48GGK-(LPT)-H_6 with Ub(LAT) in the presence of Srt2A leads to formation of the desired branched triUb and both diUb intermediates. '**' denotes impurity present in Srt2A. **c)** Purification of the K11/K48 branched triUb using Ni-NTA chromatography followed by SEC (Superdex 75 16/600). Ni-NTA: Ni-Nitrilotriacetic acid, FT: flowthrough, Elu: eluant.

Yields of sortylation were determined densitometrically using ImageJ from Supplementary Figure 13b: K11/K48, 1 h, 54 %.

Consistent results were obtained over at least three replicate experiments.

Source data are provided as a Source Data file.

Supplementary Tables

Supplementary Table 1. Primers for introduction of TAG mutants

Construct	Primer	Sequence (5‘ => 3‘)
**pPylT_SUMO2-K11TAG-H$_6$**	Short Fw	ACCGAAAACAACGATCATATTAACCTGAAAGT
	Short Rv	TTCATCCGCCATGGTTAATTCCTCCT
	Tail Fw	AAACCGAAAGAAGGCGTGTAGACCGAAAACAACGATCATATTAACCTGAAAGT
	Tail Rv	CTACACGCCTTCTTTCGGTTTTTCATCCGCCATGGTTAATTCCTCCT
**pPylT_SUMO2-K21TAG-H$_6$**	Short Fw	GTGGCGGGCCAGGATG
	Short Rv	ATGATCGTTGTTTTCGGTCTACACGC
	Tail Fw	ATTAACCTGTAGGTGGCGGGCCAGGATG
	Tail Rv	CTACAGGTTAATATGATCGTTGTTTTCGGTCTACACGC
**pPylT_SUMO2-K33TAG-H$_6$**	Short Fw	CATACCCCGCTGAGCAAACTGA
	Short Rv	AAACTGCACCACGCTGCCA
	Tail Fw	TAGATTAAACGCCATACCCCGCTGAGCAAACTGA
	Tail Rv	GCGTTTAATCTAAAACTGCACCACGCTGCCA
**pPylT_SUMO2-K35TAG-H$_6$**	Short Fw	CATACCCCGCTGAGCAAACTGA
	Short Rv	AAACTGCACCACGCTGCCA
	Tail Fw	AAAATTTAGCGCCATACCCCGCTGAGCAAACTGA
	Tail Rv	GCGCTAAATTTTAAACTGCACCACGCTGCCA
**pPylT_SUMO2-K42TAG-H$_6$**	Short Fw	GCGTATTGCGAACGCCAGG
	Short Rv	GCTCAGCGGGGTATGGCG
	Tail Fw	TAGCTGATGAAAGCGTATTGCGAACGCCAGG
	Tail Rv	TTTCATCAGCTAGCTCAGCGGGGTATGGCG
**pPylT_SUMO2-K45TAG-H$_6$**	Short Fw	GCGTATTGCGAACGCCAGG
	Short Rv	GCTCAGCGGGGTATGGCG
	Tail Fw	AAACTGATGTAGGCGTATTGCGAACGCCAGG
	Tail Rv	CTACATCAGTTTGCTCAGCGGGGTATGGCG
**pPylT_Ub-K6TAG-H$_6$**	Short Fw	ACCATCACTCTCGAAGTGGAGC
	Short Rv	CACGAAGATCTGCATGGTTAATTCCTCC
	Tail Fw	TAGACCCTGACTGGTAAGACCATCACTCTCGAAGTGGAGC
	Tail Rv	CTTACCAGTCAGGGTCTACACGAAGATCTGCATGGTTAATTCCTCC
**pPylT_Ub-K11TAG-H$_6$**	Short Fw	ACCATCACTCTCGAAGTGGAGC
	Short Rv	CACGAAGATCTGCATGGTTAATTCCTCC
	Tail Fw	AAGACCCTGACTGGTTAGACCATCACTCTCGAAGTGGAGC

	Tail Rv	CTAACCAGTCAGGGTCTTCACGAAGATCTGCATGGTTAATTCCTCC
**pPylT_Ub-K6/11TAG-H_6**	Short Fw	ACCATCACTCTCGAAGTGGAGC
	Short Rv	CACGAAGATCTGCATGGTTAATTCCTCC
	Tail Fw	TAGACCCTGACTGGTTAGACCATCACTCTCGAAGTGGAGC
	Tail Rv	CTAACCAGTCAGGGTCTACACGAAGATCTGCATGGTTAATTCCTCC
**pPylT_Ub-K48TAG-H_6**	Short Fw	CAGCTGGAAGATGGACGCAC
	Short Rv	GATCAACCTCTGCTGGTCAGGAG
	Tail Fw	TTTGCTGGGTAGCAGCTGGAAGATGGACGCAC
	Tail Rv	CTACCCAGCAAAGATCAACCTCTGCTGGTCAGGAG
**pPylT_Ub-K63TAG-H_6**	Short Fw	CTGCACCTGGTCCTCCGTCTCA
	Short Rv	CTGGATGTTGTAGTCAGACAGGGT
	Tail Fw	TAGGAGTCCACCCTGCACCTGGTCCTCCGT
	Tail Rv	GGTGGACTCCTACTGGATGTTGTAGTCAGA

Supplementary Table 2. Primers for introduction of sortase motifs

Construct	Primer	Sequence (5‘ => 3‘)
**pET17b_Ub(PS)**	Short Fw	GGTGGGTGATAAGAATTCTGCAGATATCC
	Short Rv	GACCAGGTGCAGGGTGGAC
	Tail Fw	CTCCCGCTCAGCGGTGGGTGATAAGAATTCTGCAGATATCC
	Tail Rv	GCTGAGCGGGAGGACCAGGTGCAGGGTGGAC
**pET17b_Ub(LPS)**	Short Fw	GGTGGGTGATAAGAATTCTGCAGATATCC
	Short Rv	GACCAGGTGCAGGGTGGAC
	Tail Fw	CTGCTCCCGCTCAGCGGTGGGTGATAAGAATTCTGCAGATATCC
	Tail Rv	GCTGAGCGGGAGCAGGACCAGGTGCAGGGTGGAC
**pET17b_Ub(PT)**	Short Fw	GGTGGGTGATAAGAATTCTGCAGATATCC
	Short Rv	GACCAGGTGCAGGGTGGAC
	Tail Fw	CTCCCGCTCACCGGTGGGTGATAAGAATTCTGCAGATATCC
	Tail Rv	GGTGAGCGGGAGGACCAGGTGCAGGGTGGAC
**pET17b_Ub(LPT)**	Short Fw	GGTGGGTGATAAGAATTCTGCAGATATCC
	Short Rv	GACCAGGTGCAGGGTGGAC
	Tail Fw	CTGCTCCCGCTCACCGGTGGGTGATAAGAATTCTGCAGATATCC
	Tail Rv	GGTGAGCGGGAGCAGGACCAGGTGCAGGGTGGAC
**pET17b_Ub(AT)**	Short Fw	GGTGGGTGATAAGAATTCTGCAGATATCC
	Short Rv	GACCAGGTGCAGGGTGGAC
	Tail Fw	CTCGCTCTCACCGGTGGGTGATAAGAATTCTGCAGATATCC
	Tail Rv	GGTGAGAGCGAGGACCAGGTGCAGGGTGGAC
**pET17b_Ub(LAT)**	Short Fw	GGTGGGTGATAAGAATTCTGCAGATATCC
	Short Rv	GACCAGGTGCAGGGTGGAC
	Tail Fw	CTGCTCGCTCTCACCGGTGGGTGATAAGAATTCTGCAGATATCC
	Tail Rv	GGTGAGAGCGAGCAGGACCAGGTGCAGGGTGGAC

Supplementary Table 3. Plasmids

Plasmid	Description
**pBK_AzGGKRS**	*M. bakeri* AzGGK aminoacyl-tRNA-synthetase (aaRS) under a constitutive GlnS promotor.
**pBK_wt_RS**	*M. bakeri* wt aaRS under a constitutive GlnS promotor.
**pPylT_sfGFP-N150TAG-H_6**	sfGFP-N150TAG-H_6 under an arabinose promotor with a C-terminal H_6-tag and a PylT copy under a constitutive promotor.
**pPylT_UbK6TAG-H_6**	UbK6TAG-H_6 under an arabinose promotor with a C-terminal H_6-tag and a PylT copy under a constitutive promotor.
**pPylT_UbK48TAG-H_6**	UbK48TAG-H_6 under an arabinose promotor with a C-terminal H_6-tag and a PylT copy under a constitutive promotor.
**pPylT_UbK48TAG-(LPT)-H_6**	UbK48TAG-H_6 with LLPLTGG C-terminus followed by a C-terminal H_6-tag under an arabinose promotor and a PylT copy under a constitutive promotor.
**pPylT_UbK63TAG-(PT)-H_6**	UbK63TAG-H_6 with LPLTGG C-terminus under an arabinose promotor with a C-terminal H_6-tag and a PylT copy under a constitutive promotor.
**pPylT_UbK63TAG-(LPT)-H_6**	UbK63TAG-H_6 with LLPLTGG C-terminus followed by a C-terminal H_6-tag under an arabinose promotor and a PylT copy under a constitutive promotor.
**pPylT_Ub-KxxTAG-KyyTAG-H_6**	UbKxxTAG-KyyTAG with a C-terminal H_6-tag under an arabinose promotor and a PylT copy under a constitutive promotor.
**pPylT_SUMO2-KxxTAG-H_6**	SUMO2KxxTAG with a C-terminal H_6-tag under an arabinose promotor and a PylT copy under a constitutive promotor.
**pET29b_Srt4S-H_6**	Srt4S under an IPTG inducible T7 Promotor with a C-terminal H_6-tag.
**pET29b_Srt5M-H_6**	Srt5M under an IPTG inducible T7 Promotor with a C-terminal H_6-tag.
**pET29b_Srt2A-H_6**	Srt2A under an IPTG inducible T7 Promotor with a C-terminal H_6-tag.
**pET29b_Srt4S-TEV-H_6**	Srt4S with a C-terminal TEV-site followed by a H_6-tag under an IPTG inducible T7 Promotor with a C-terminal H_6-tag.
**pET29b_Srt5M-TEV-H_6**	Srt5M with a C-terminal TEV-site followed by a H_6-tag under an IPTG inducible T7 Promotor with a C-terminal H_6-tag.

**pET29b_Srt2A-TEV-H_6**	Srt2A with a C-terminal TEV-site followed by a H_6-tag under an IPTG inducible T7 Promotor with a C-terminal H_6-tag.
**pET17b_H_6-Rap80(1-137)_7A_Linker**	Rap80(1-137)_7A_Linker with an C-terminal H_6-tag under an IPTG inducible T7 Promotor (used for pull-down experiments).
**pET17b_H_6-Rap80(35-124)_7A_Linker_C70S_C120S**	Rap80(35-124)_7A_Linker with C70S/C120S mutations and an C-terminal H_6-tag under an IPTG inducible T7 Promotor (used for fluorescence anisotropy experiments).
**pET17b_H_6-Rap80(35-124)_7A_Linker**	Rap80(35-124)_7A_Linker with an C-terminal H_6-tag under an IPTG inducible T7 Promotor (used for NMR experiments).
**pET17b_H_6-TEV-G-SUMO2**	SUMO2 with an N-terminal H_6-tag followed by a TEV site and an additional glycine under an IPTG inducible T7 Promotor.
**pET17b_Ub_wt**	Ubiquitin wt under an IPTG inducible T7 Promotor.
**pET17b_Ub_wt-H_6**	Ubiquitin wt with C-terminal H_6-tag under an IPTG inducible T7 Promotor.
**pET17b_Ub(PS)**	Ubiquitin with C-terminus LPLSGG under an IPTG inducible T7 Promotor.
**pET17b_Ub(LPS)**	Ubiquitin with C-terminus LLPLSGG under an IPTG inducible T7 Promotor.
**pET17b_Ub(PT)**	Ubiquitin with C-terminus LPLTGG under an IPTG inducible T7 Promotor.
**pET17b_Ub(PT)-H_6**	Ubiquitin with C-terminus LPLTGG followed by an H_6-tag under an IPTG inducible T7 Promotor.
**pET17b_Ub(LPT)**	Ubiquitin with C-terminus LLPLTGG under an IPTG inducible T7 Promotor.
**pET17b_Ub(LPT)-H_6**	Ubiquitin with C-terminus LLPLTGG followed by an H_6-tag under an IPTG inducible T7 Promotor.
**pET17b_Ub(AT)**	Ubiquitin with C-terminus LALTGG under an IPTG inducible T7 Promotor.
**pET17b_Ub(AT)-H_6**	Ubiquitin with C-terminus LALTGG followed by an H_6-tag under an IPTG inducible T7 Promotor.
**pET17b_Ub(LAT)**	Ubiquitin with C-terminus LLALTGG under an IPTG inducible T7 Promotor.

**pET17b_Ub(LAT)-H_6**	Ubiquitin with C-terminus LLALTGG followed by an H_6-tag under an IPTG inducible T7 Promotor.
**pET17b_Ub(LAT*)-H_6**	Ubiquitin with C-terminus LLALTGYEAAAK followed by a H_6-tag under an IPTG inducible T7 Promotor.
**pET17b_Ub(LPT*)-H_6**	Ubiquitin with C-terminus LLPLTGYEAAAK followed by a H_6-tag under an IPTG inducible T7 Promotor.
**pET17b_Ub(LPT*H)-H_6**	Ubiquitin with C-terminus LLPLTGLHGYEAAAK followed by a H_6-tag under an IPTG inducible T7 Promotor.
**pGEX-6P-1-UBE2R1**	GST-UBE2R1 (Cdc34) under an IPTG inducible T7 Promotor.
**pGEX-6P-1-UBE2N**	GST-UBE2N (Ubc13) under an IPTG inducible T7 Promotor
**pGEX-6P-1-UBE2V1**	GST-UBE2V1 (Uev1A) under an IPTG inducible T7 Promotor
**pET28a_H_6-Thr-USP2**	Catalytic domain of USP2 with an N-terminal H_6-tag followed by a thrombin cleavage site under an IPTG inducible T7 Promotor.
**pET28a_H_6-Thr-SENP2**	Catalytic domain of SENP2 H_6-tag followed by a thrombin cleavage site under an IPTG inducible T7 Promotor.
**pET28a_H_6-Thr-UCHL3**	UCHL3 with an N-terminal H_6-tag followed by a thrombin cleavage site under an IPTG inducible T7 Promotor

Supplementary Notes

Supplementary Note 1: Amino acid sequences of proteins

AzGGKRS (mutations: L274A, N311Q, C313S)
MDKKPLDVLISATGLWMSRTGTLHKIKHHEVSRSKIYIEMACGDHLVVNNSRSCRTARAFRHHKYRKT
CKRCRVSDEDINNFLTRSTESKNSVKVRVVSAPKVKKAMPKSVSRAPKPLENSVSAKASTNTSRSVPSP
AKSTPNSSVPASAPAPSLTRSQLDRVEALLSPEDKISLNMAKPFRELEPELVTRRKNDFQRLYTNDREDY
LGKLERDITKFFVDRGFLEIKSPILIPAEYVERMGINNDTELSKQIFRVDKNLCLRPMLAPTLYNY**A**RKLD
RILPGPIKIFEVGPCYRKESDGKEHLEEFTMV**Q**F**S**QMGSGCTRENLEALIKEFLDYLEIDFEIVGDSCMVY
GDTLDIMHGDLELSSAVVGPVSLDREWGIDKPWIGAGFGLERLLKVMHGFKNIKRASRSESYYNGISTN
L*

wt RS
MDKKPLDVLISATGLWMSRTGTLHKIKHHEVSRSKIYIEMACGDHLVVNNSRSCRTARAFRHHKYRKT
CKRCRVSDEDINNFLTRSTESKNSVKVRVVSAPKVKKAMPKSVSRAPKPLENSVSAKASTNTSRSVPSP
AKSTPNSSVPASAPAPSLTRSQLDRVEALLSPEDKISLNMAKPFRELEPELVTRRKNDFQRLYTNDREDY
LGKLERDITKFFVDRGFLEIKSPILIPAEYVERMGINNDTELSKQIFRVDKNLCLRPMLAPTLYNYLRKLD
RILPGPIKIFEVGPCYRKESDGKEHLEEFTMVNFCQMGSGCTRENLEALIKEFLDYLEIDFEIVGDSCMVY
GDTLDIMHGDLELSSAVVGPVSLDREWGIDKPWIGAGFGLERLLKVMHGFKNIKRASRSESYYNGISTN
L*

sfGFP-N150TAG-H$_6$
MPSKGEELFTGVVPILVELDGDVNGHKFSVRGEGEGDATNGKLTLKFICTTGKLPVPWPTLVTTLTYGV
QCFSRYPDHMKRHDFFKSAMPEGYVQERTISFKDDGTYKTRAEVKFEGDTLVNRIELKGIDFKEDGNIL
GHKLEYNFNSH*VYITADKQKNGIKANFKIRHNVEDGSVQLADHYQQNTPIGDGPVLLPDNHYLSTQS
VLSKDPNEKRDHMVLLEFVTAAGITHGMDELYKGSHHHHHH

Ubiquitin-K6TAG-H$_6$
MQIFV*TLTGKTITLEVEPSDTIENVKAKIQDKEGIPPDQQRLIFAGKQLEDGRTLSDYNIQKESTLHLVL
RLRGGHHHHHH*

Ubiquitin-K48TAG-H$_6$
MQIFVKTLTGKTITLEVEPSDTIENVKAKIQDKEGIPPDQQRLIFAG*QLEDGRTLSDYNIQKESTLHLVL
RLRGGHHHHHH*

Ubiquitin-K48TAG-(LPT)-H$_6$
MQIFVKTLTGKTITLEVEPSDTIENVKAKIQDKEGIPPDQQRLIFAG*QLEDGRTLSDYNIQKESTLHLVLL
PLTGGHHHHHH*

Ubiquitin-K63TAG-(PT)-H$_6$
MQIFVKTLTGKTITLEVEPSDTIENVKAKIQDKEGIPPDQQRLIFAGKQLEDGRTLSDYNIQ*ESTLHLVLP
LTGGHHHHHH*

Ubiquitin-K63TAG-(LPT)-H$_6$
MQIFVKTLTGKTITLEVEPSDTIENVKAKIQDKEGIPPDQQRLIFAGKQLEDGRTLSDYNIQ*ESTLHLVLL
PLTGGHHHHHH*

Ubiquitin-K48TAG-K63TAG-H$_6$
MQIFVKTLTGKTITLEVEPSDTIENVKAKIQDKEGIPPDQQRLIFAG*QLEDGRTLSDYNIQ*ESTLHLVLR
LRGGHHHHHH*

Ubiquitin-K6TAG-K63TAG-H$_6$
MQIFV*TLTGKTITLEVEPSDTIENVKAKIQDKEGIPPDQQRLIFAGKQLEDGRTLSDYNIQ*ESTLHLVLR
LRGGHHHHHH*

Ubiquitin-K6TAG-K48TAG-H$_6$
MQIFV*TLTGKTITLEVEPSDTIENVKAKIQDKEGIPPDQQRLIFAG*QLEDGRTLSDYNIQKESTLHLVLR
LRGGHHHHHH*

Ubiquitin-K11TAG-K63TAG-H_6
MQIFVKTLTG*TITLEVEPSDTIENVKAKIQDKEGIPPDQQRLIFAGKQLEDGRTLSDYNIQ*ESTLHLVLR
LRGGHHHHHH*

Ubiquitin-K11TAG-K48TAG-H_6
MQIFVKTLTG*TITLEVEPSDTIENVKAKIQDKEGIPPDQQRLIFAG*QLEDGRTLSDYNIQKESTLHLVLR
LRGGHHHHHH*

Ubiquitin-K6TAG-K11TAG-H_6
MQIFV*TLTG*TITLEVEPSDTIENVKAKIQDKEGIPPDQQRLIFAGKQLEDGRTLSDYNIQKESTLHLVLR
LRGGHHHHHH*

Ubiquitin-K11TAG-K48TAG-(LPT)-H_6
MQIFVKTLTG*TITLEVEPSDTIENVKAKIQDKEGIPPDQQRLIFAG*QLEDGRTLSDYNIQKESTLHLVLL
PLTGGHHHHHH*

SUMO2-K5TAG-H_6
MADE*PKEGVKTENNDHINLKVAGQDGSVVQFKIKRHTPLSKLMKAYCERQGLSMRQIRFRFDGQPIN
ETDTPAQLEMEDEDTIDVFQQQTGGHHHHHH*

SUMO2-K7TAG-H_6
MADEKP*EGVKTENNDHINLKVAGQDGSVVQFKIKRHTPLSKLMKAYCERQGLSMRQIRFRFDGQPIN
ETDTPAQLEMEDEDTIDVFQQQTGGHHHHHH*

SUMO2-K11TAG-H_6
MADEKPKEGV*TENNDHINLKVAGQDGSVVQFKIKRHTPLSKLMKAYCERQGLSMRQIRFRFDGQPIN
ETDTPAQLEMEDEDTIDVFQQQTGGHHHHHH*

SUMO2-K21TAG-H_6
MADEKPKEGVKTENNDHINL*VAGQDGSVVQFKIKRHTPLSKLMKAYCERQGLSMRQIRFRFDGQPIN
ETDTPAQLEMEDEDTIDVFQQQTGGHHHHHH*

SUMO2-K33TAG-H_6
MADEKPKEGVKTENNDHINLKVAGQDGSVVQF*IKRHTPLSKLMKAYCERQGLSMRQIRFRFDGQPIN
ETDTPAQLEMEDEDTIDVFQQQTGGHHHHHH*

SUMO2-K35TAG-H_6
MADEKPKEGVKTENNDHINLKVAGQDGSVVQFKI*RHTPLSKLMKAYCERQGLSMRQIRFRFDGQPIN
ETDTPAQLEMEDEDTIDVFQQQTGGHHHHHH*

SUMO2-K42TAG-H_6
MADEKPKEGVKTENNDHINLKVAGQDGSVVQFKIKRHTPLS*LMKAYCERQGLSMRQIRFRFDGQPIN
ETDTPAQLEMEDEDTIDVFQQQTGGHHHHHH*

SUMO2-K45TAG-H_6
MADEKPKEGVKTENNDHINLKVAGQDGSVVQFKIKRHTPLSKLM*AYCERQGLSMRQIRFRFDGQPIN
ETDTPAQLEMEDEDTIDVFQQQTGGHHHHHH*

Srt4S-H_6
MQAKPQIPKDKSKVAGYIEIPDADIKEPVYPGPATREQLDRGVCFVEENESLDDQNISITGHTAIDRPNY
QFTNLRAAKKGSMVYLKVGNETRKYKMTSIRNVKPTAVEVLDEQKGKDKQLTLVTCDDYNFETGVW
ETRKIFVATEVKGSHHHHHH*

Srt5M-H_6
MQAKPQIPKDKSKVAGYIEIPDADIKEPVYPGPATREQLNRGVSFAEENESLDDQNISIAGHTFIDRPNYQ
FTNLKAAKKGSMVYFKVGNETRKYKMTSIRNVKPTAVEVLDEQKGKDKQLTLITCDDYNEETGVWET
RKIFVATEVKLEHHHHHH*

Srt2A-H_6

MQAKPQIPKDKSKVAGYIEIPDADIKEPVYPGPATREQLNRGVCFHDENESLDDQNISIAGHTFIDRPNY
QFTNLKAAKPGSMVYFKVGNETRIYKMTSIRKVHPNAVEVLDEQEGKDKQLTLVTCDDYNEETGVWE
SRKIFVATEVKGSHHHHHH*

Srt4S-TEV-H_6
MQAKPQIPKDKSKVAGYIEIPDADIKEPVYPGPATREQLDRGVCFVEENESLDDQNISITGHTAIDRPNY
QFTNLRAAKKGSMVYLKVGNETRKYKMTSIRNVKPTAVEVLDEQKGKDKQLTLVTCDDYNFETGVW
ETRKIFVATEVKGSENLYFQGHHHHHH*

Srt5M-TEV-H_6
MQAKPQIPKDKSKVAGYIEIPDADIKEPVYPGPATREQLNRGVSFAEENESLDDQNISIAGHTFIDRPNYQ
FTNLKAAKKGSMVYFKVGNETRKYKMTSIRNVKPTAVEVLDEQKGKDKQLTLITCDDYNEETGVWET
RKIFVATEVKLEENLYFQGHHHHHH*

Srt2A-TEV-H_6
MQAKPQIPKDKSKVAGYIEIPDADIKEPVYPGPATREQLNRGVCFHDENESLDDQNISIAGHTFIDRPNY
QFTNLKAAKPGSMVYFKVGNETRIYKMTSIRKVHPNAVEVLDEQEGKDKQLTLVTCDDYNEETGVWE
SRKIFVATEVKGSENLYFQGHHHHHH*

H_6-Rap80(1-137) 7A Linker
MPSSHHHHHHSSGPRRKKKVKEVSESRNLEKKDVETTSSVSVKRKRRLEDAFIVISDSDGEEPKEENGL
QKTKTKQSNRAKCLAKRKIAQMTEEEQFALALKMSEQEAAAAAAAAEEEEELLRKAIAESLNSCRPSD
ASATRSRPLATG*

H_6-Rap80(35-124) 7A Linker C70S C120S

MPSCHHHHHHSSGRLEDAFIVISDSDGEEPKEENGLQKTKTKQSNRAKSLAKRKIAQMTEEEQFALALK
MSEQEAAAAAAAAEEEEELLRKAIAESLNSSRPS*

H_6-Rap80(35-124) 7A Linker

MPSSHHHHHHSSGRLEDAFIVISDSDGEEPKEENGLQKTKTKQSNRAKCLAKRKIAQMTEEEQFALALK
MSEQEAAAAAAAAEEEEELLRKAIAESLNSCRPS*

H_6-TEV-G-SUMO2
MHHHHHHENLYFQGGADEKPKEGVKTENNDHINLKVAGQDGSVVQFKIKRHTPLSKLMKAYCERQG
LSMRQIRFRFDGQPINETDTPAQLEMEDEDTIDVFQQQTGG*

wt Ubiquitin
MQIFVKTLTGKTITLEVEPSDTIENVKAKIQDKEGIPPDQQRLIFAGKQLEDGRTLSDYNIQKESTLHLVL
RLRGG*

wt Ubiquitin-H_6
MQIFVKTLTGKTITLEVEPSDTIENVKAKIQDKEGIPPDQQRLIFAGKQLEDGRTLSDYNIQKESTLHLVL
RLRGGHHHHHH*

Ub(PS)
MQIFVKTLTGKTITLEVEPSDTIENVKAKIQDKEGIPPDQQRLIFAGKQLEDGRTLSDYNIQKESTLHLVL
PLSGG*

Ub(LPS)
MQIFVKTLTGKTITLEVEPSDTIENVKAKIQDKEGIPPDQQRLIFAGKQLEDGRTLSDYNIQKESTLHLVL
LPLSGG*

Ub(PT)
MQIFVKTLTGKTITLEVEPSDTIENVKAKIQDKEGIPPDQQRLIFAGKQLEDGRTLSDYNIQKESTLHLVL
PLTGG*

Ub(PT)-H_6
MQIFVKTLTGKTITLEVEPSDTIENVKAKIQDKEGIPPDQQRLIFAGKQLEDGRTLSDYNIQKESTLHLVL
PLTGGHHHHHH*

Ub(LPT)

MQIFVKTLTGKTITLEVEPSDTIENVKAKIQDKEGIPPDQQRLIFAGKQLEDGRTLSDYNIQKESTLHLVL
LPLTGG*

Ub(LPT)-H_6
MQIFVKTLTGKTITLEVEPSDTIENVKAKIQDKEGIPPDQQRLIFAGKQLEDGRTLSDYNIQKESTLHLVL
LPLTGGHHHHHH*

Ub(AT)
MQIFVKTLTGKTITLEVEPSDTIENVKAKIQDKEGIPPDQQRLIFAGKQLEDGRTLSDYNIQKESTLHLVL
ALTGG*

Ub(AT)-H_6
MQIFVKTLTGKTITLEVEPSDTIENVKAKIQDKEGIPPDQQRLIFAGKQLEDGRTLSDYNIQKESTLHLVL
ALTGGHHHHHH*

Ub(LAT)
MQIFVKTLTGKTITLEVEPSDTIENVKAKIQDKEGIPPDQQRLIFAGKQLEDGRTLSDYNIQKESTLHLVL
LALTGG*

Ub(LAT)-H_6
MQIFVKTLTGKTITLEVEPSDTIENVKAKIQDKEGIPPDQQRLIFAGKQLEDGRTLSDYNIQKESTLHLVL
LALTGGHHHHHH*

Ub(LAT*)
MQIFVKTLTGKTITLEVEPSDTIENVKAKIQDKEGIPPDQQRLIFAGKQLEDGRTLSDYNIQKESTLHLVL
LALTGYEAAAKHHHHHH*

Ub(LPT*)
MQIFVKTLTGKTITLEVEPSDTIENVKAKIQDKEGIPPDQQRLIFAGKQLEDGRTLSDYNIQKESTLHLVL
LPLTGYEAAAKHHHHHH*

Ub(LPT*)
MQIFVKTLTGKTITLEVEPSDTIENVKAKIQDKEGIPPDQQRLIFAGKQLEDGRTLSDYNIQKESTLHLVL
LPLTGLHGYEAAAKHHHHHH*

H_6-Thrombin-USP2
MGSSHHHHHHSSGLVPRGSSSPGRDGMNSKSAQGLAGLRNLGNTCFMNSILQCLSNTRELRDYCLQRL
YMRDLHHGSNAHTALVEEFAKLIQTIWTSSPNDVVSPSEFKTQIQRYAPRFVGYNQQDAQEFFRFLLDG
LHNEVNRVTLRPKSNPENLDHLPDDEKGRQMWRKYLEREDSRIGDLFVGQLKSSLTCTDCGYCSTVFD
PFWDLSLPIAKRGYPEVTLMDCMRLFTKEDVLDGDAAPTCCRCRGRKRCIKKFSIQRFPKILVLHLKRFS
ESRIRTSKLTTFVNFPLRDLDLREFASENTNHAVYNLYAVSNHSGTTMGGHYTAYCRSPGTGEWHTFN
DSSVTPMSSSQVRTSDAYLLFYELASPPSRM*

H_6-Thrombin-SENP2
MGSSHHHHHHSSGLVPRGSHMDLLELTEDMEKEISNALGHGPQDEILSSAFKLRITRGDIQTLKNYHWL
NDEVINFYMNLLVERNKKQGYPALHVFSTFFYPKLKSGGYQAVKRWTKGVNLFEQEIILVPIHRKVHW
SLVVIDLRKKCLKYLDSMGQKGHRICEILLQYLQDESKTKRNSDLNLLEWTHHSMKPHEIPQQLNGSDC
GMFTCKYADYISRDKPITFTQHQMPLFRKKMVWEILHQQLL*

H_6-Thrombin-UCHL3

MGSSHHHHHHSSGLVPRGSHMEGQRWLPLEANPEVTNQFLKQLGLHPNWQFVDVYGMDPELLSMVP
RPVCAVLLLFPITEKYEVFRTEEEEKIKSQGQDVTSSVYFMKQTISNACGTIGLIHAIANNKDKMHFESGS
TLKKFLEESVSMSPEERARYLENYDAIRVTHETSAHEGQTEAPSIDEKVDLHFIALVHVDGHLYELDGR
KPFPINHGETSDETLLEDAIEVCKKFMERDPDELRFNAIALSAA*

GST-UBE2R1 (Cdc34)

MSPILGYWKIKGLVQPTRLLLEYLEEKYEEHLYERDEGDKWRNKKFELGLEFPNLPYYIDGDVKLTQS
MAIIRYIADKHNMLGGCPKERAEISMLEGAVLDIRYGVSRIAYSKDFETLKVDFLSKLPEMLKMFEDRL
CHKTYLNGDHVTHPDFMLYDALDVVLYMDPMCLDAFPKLVCFKKRIEAIPQIDKYLKSSKYIAWPLQG
WQATFGGGDHPPKSDLEVLFQGPLGSARPLVPSSQKALLLELKGLQEEPVEGFRVTLVDEGDLYNWEV

AIFGPPNTYYEGGYFKARLKFPIDYPYSPPAFRFLTKMWHPNIYETGDVCISILHPPVDDPQSGELPSERW
NPTQNVRTILLSVISLLNEPNTFSPANVDASVMYRKWKESKGKDREYTDIIRKQVLGTKVDAERDGVK
VPTTLAEYCVKTKAPAPDEGSDLFYDDYYEDGEVEEEADSCFGDDEDDSGTEES*

GST-UBE2N (Ubc13)

MSPILGYWKIKGLVQPTRLLLEYLEEKYEEHLYERDEGDKWRNKKFELGLEFPNLPYYIDGDVKLTQS
MAIIRYIADKHNMLGGCPKERAEISMLEGAVLDIRYGVSRIAYSKDFETLKVDFLSKLPEMLKMFEDRL
CHKTYLNGDHVTHPDFMLYDALDVVLYMDPMCLDAFPKLVCFKKRIEAIPQIDKYLKSSKYIAWPLQG
WQATFGGGDHPPKSDLEVLFQGPLGSAGLPRRIIKETQRLLAEPVPGIKAEPDESNARYFHVVIAGPQDS
PFEGGTFKLELFLPEEYPMAAPKVRFMTKIYHPNVDKLGRICLDILKDKWSPALQIRTVLLSIQALLSAP
NPDDPLANDVAEQWKTNEAQAIETARAWTRLYAMNNI*

GST-UBE2V1 (Uev1A)

MSPILGYWKIKGLVQPTRLLLEYLEEKYEEHLYERDEGDKWRNKKFELGLEFPNLPYYIDGDVKLTQS
MAIIRYIADKHNMLGGCPKERAEISMLEGAVLDIRYGVSRIAYSKDFETLKVDFLSKLPEMLKMFEDRL
CHKTYLNGDHVTHPDFMLYDALDVVLYMDPMCLDAFPKLVCFKKRIEAIPQIDKYLKSSKYIAWPLQG
WQATFGGGDHPPKSDLEVLFQGPLGSPGEVQASYLKSQSKLSDEGRLEPRKFHCKGVKVPRNFRLLEE
LEEGQKGVGDGTVSWGLEDDEDMTLTRWTGMIIGPPRTIYENRIYSLKIECGPKYPEAPPFVRFVTKIN
MNGVNSSNGVVDPRAISVLAKWQNSYSIKVVLQELRRLMMSKENMKLPQPPEGQCYSN*

Supplementary References

1. Kulathu, Y., Akutsu, M., Bremm, A., Hofmann, K. & Komander, D. Two-sided ubiquitin binding explains specificity of the TAB2 NZF domain. *Nat Struct Mol Biol* **16**, 1328-30 (2009).
2. Varadan, R., Assfalg, M., Raasi, S., Pickart, C. & Fushman, D. Structural determinants for selective recognition of a Lys48-linked polyubiquitin chain by a UBA domain. *Mol Cell* **18**, 687-98 (2005).
3. Reed, S.A., Brzovic, D.A., Takasaki, S.S., Boyko, K.V. & Antos, J.M. Efficient Sortase-Mediated Ligation Using a Common C-Terminal Fusion Tag. *Bioconjug Chem* **31**, 1463-1473 (2020).
4. Sims, J.J. & Cohen, R.E. Linkage-specific avidity defines the lysine 63-linked polyubiquitin-binding preference of rap80. *Mol Cell* **33**, 775-83 (2009).
5. Anamika & Spyracopoulos, L. Molecular Basis for Phosphorylation-dependent SUMO Recognition by the DNA Repair Protein RAP80. *J Biol Chem* **291**, 4417-28 (2016).
6. Shen, Y., Delaglio, F., Cornilescu, G. & Bax, A. TALOS+: a hybrid method for predicting protein backbone torsion angles from NMR chemical shifts. *J Biomol NMR* **44**, 213-23 (2009).
7. Kyte, J. & Doolittle, R.F. A simple method for displaying the hydropathic character of a protein. *J Mol Biol* **157**, 105-32 (1982).
