# Supplementary material for: A modular toolbox to generate complex polymeric ubiquitin architectures using orthogonal sortase enzymes
Source: Nat Commun. 2021 Nov 11;12:6515. doi: 10.1038/s41467-021-26812-9 (PMC8585875; doi:10.1038/s41467-021-26812-9)
Supplement: Supplementary file 3 — Reporting Summary [file 41467_2021_26812_MOESM3_ESM.pdf]

nature portfolio

Corresponding author(s): Kathrin Lang

Last updated by author(s): 23.09.2021

Reporting Summary

Nature Portfolio wishes to improve the reproducibility of the work that we publish. This form provides structure for consistency and transparency in reporting. For further information on Nature Portfolio policies, see our Editorial Policies and the Editorial Policy Checklist.

Statistics

For all statistical analyses, confirm that the following items are present in the figure legend, table legend, main text, or Methods section.

n/a	Confirmed	
☐	☒	The exact sample size (n) for each experimental group/condition, given as a discrete number and unit of measurement
☐	☒	A statement on whether measurements were taken from distinct samples or whether the same sample was measured repeatedly
☒	☐	The statistical test(s) used AND whether they are one- or two-sided<br>*Only common tests should be described solely by name; describe more complex techniques in the Methods section.*
☒	☐	A description of all covariates tested
☒	☐	A description of any assumptions or corrections, such as tests of normality and adjustment for multiple comparisons
☐	☒	A full description of the statistical parameters including central tendency (e.g. means) or other basic estimates (e.g. regression coefficient) AND variation (e.g. standard deviation) or associated estimates of uncertainty (e.g. confidence intervals)
☒	☐	For null hypothesis testing, the test statistic (e.g. F, t, r) with confidence intervals, effect sizes, degrees of freedom and P value noted<br>*Give P values as exact values whenever suitable.*
☒	☐	For Bayesian analysis, information on the choice of priors and Markov chain Monte Carlo settings
☒	☐	For hierarchical and complex designs, identification of the appropriate level for tests and full reporting of outcomes
☒	☐	Estimates of effect sizes (e.g. Cohen's d, Pearson's r), indicating how they were calculated

Our web collection on statistics for biologists contains articles on many of the points above.

Software and code

Policy information about availability of computer code

Data collection	Jasco Fluorescence Spectrometer FP-8500: SpectraManager II<br>Agilent Technologies 1260 Infinity LC-MS 6310 Quadrupole spectrometer: OpenLAB CDS ChemStation Edition for LC-MS, Rev. C.01.07 SR3<br>Bruker Avance III 900 MHz spectrometer: Topspin 3.5 Pl 6
Data analysis	Kaleidagraph software, version 4.5 (Synergy, Reading, UK)<br>Prism 9.2.0 (GraphPad Software, LLC)<br>protparam (https://web.expasy.org/protparam/)<br>ImageJ 1.49v, NIH, http://imahej.nih.gov/ij<br>NMRPipe Linux64: 20210129<br>CcpNMR Analysis version 2.4.2

For manuscripts utilizing custom algorithms or software that are central to the research but not yet described in published literature, software must be made available to editors and reviewers. We strongly encourage code deposition in a community repository (e.g. GitHub). See the Nature Portfolio guidelines for submitting code & software for further information.

Data

Policy information about availability of data

All manuscripts must include a data availability statement. This statement should provide the following information, where applicable:

- Accession codes, unique identifiers, or web links for publicly available datasets
- A description of any restrictions on data availability
- For clinical datasets or third party data, please ensure that the statement adheres to our policy

The data generated or analyzed during this study are included in this article (and its supplementary information files) or are available from the corresponding author upon reasonable request. NMR data were deposited at BMRB with ID 51092. Protein structures and models used for the figures are available under the accession codes: 2N9E [https://www.rcsb.org/structure/2N9E], 2RR9 [https://www.rcsb.org/structure/2RR9], 2WWZ [https://www.rcsb.org/structure/2WWZ] and 1ZO6 [https://www.modelarchive.org/doi/10.5452/ma-cahen]. Source data are provided with this paper.

Field-specific reporting

Please select the one below that is the best fit for your research. If you are not sure, read the appropriate sections before making your selection.

[x] Life sciences   [ ] Behavioural & social sciences   [ ] Ecological, evolutionary & environmental sciences

For a reference copy of the document with all sections, see nature.com/documents/nr-reporting-summary-flat.pdf

Life sciences study design

All studies must disclose on these points even when the disclosure is negative.

Sample size	No Statistcal methods were used to pre-determine sample size. For determination of binding constants all samples were tested in 3 biological replicates.
Data exclusions	no data was excluded
Replication	For determination of binding constants all samples were tested in 3 biological replicates. All attempts at replication were successful.
Randomization	No specific attempts to randomize samples were made.
Blinding	No specific attempts for blinding were made.

Reporting for specific materials, systems and methods

We require information from authors about some types of materials, experimental systems and methods used in many studies. Here, indicate whether each material, system or method listed is relevant to your study. If you are not sure if a list item applies to your research, read the appropriate section before selecting a response.

Materials & experimental systems

n/a	Involved in the study	
[ ]	[x]	Antibodies
[x]	[ ]	Eukaryotic cell lines
[x]	[ ]	Palaeontology and archaeology
[x]	[ ]	Animals and other organisms
[x]	[ ]	Human research participants
[x]	[ ]	Clinical data
[x]	[ ]	Dual use research of concern

Methods

n/a	Involved in the study	
[x]	[ ]	ChIP-seq
[x]	[ ]	Flow cytometry
[x]	[ ]	MRI-based neuroimaging

Antibodies

Antibodies used	anti-K63: Anti-Ubiquitin (linkage specific K63) antibody (Abcam, Cat. No. ab179434), dilution 1:5,000<br>anti-mouse secondary antibody (Sigma Aldrich, Cat. No. A4416), dilution 1:5,000

Validation	All antibodies were sourced commercially.<br><br>anti-K63 antibody from Abcam was validated by the supplier (Positive control<br>WB: K63-linked-Ub2-7 recombinant protein. HEk-293 and HeLa cell lysate. Mouse and rat brain lysate. IHC: Human kidney carcinoma tissue. Human tonsil tissue. Flow Cyt (intra): HeLa cells.)
